# Supplementary material for: Allotrope-dependent activity-stability relationships of molybdenum sulfide hydrogen evolution electrocatalysts
Source: Nat Commun. 2024 Apr 29;15:3601. doi: 10.1038/s41467-024-47524-w (PMC11058198; doi:10.1038/s41467-024-47524-w)
Supplement: Supplementary file 1 — Supplementary Information [file 41467_2024_47524_MOESM1_ESM.pdf]

Supplementary Information

Allotrope-dependent activity-stability relationships of molybdenum sulfide hydrogen evolution electrocatalysts

Daniel Escalera-López[1], Christian Iffelsberger[2], Matej Zlatar[1,3], Katarina Novčić[2], Nik Maselj[4,5], Chuyen Van Pham[1], Primož Jovanovič[4,5], Nejc Hodnik[4,5], Simon Thiele[1,3], Martin Pumera[2,6,7,8], Serhiy Cherevko[1*]*

[1]Helmholtz-Institute Erlangen-Nürnberg for Renewable Energy (IEK-11), Forschungszentrum Jülich GmbH, Erlangen, Germany
Cauerstrasse 1, 91058 Erlangen, Germany

[2]Future Energy and Innovation Technology, Central European Institute of Technology, Brno University of Technology, Purkiňova 656/123, 61200 Brno, Czech Republic

[3]Department of Chemical and Biological Engineering, Friedrich-Alexander-Universität Erlangen-Nürnberg, Cauerstrasse 1, 91058 Erlangen, Germany

[4]Department of Materials Chemistry, National Institute of Chemistry, Hajdrihova 19, 1000 Ljubljana, Slovenia

[5]Faculty of Chemistry and Chemical Technology, University of Ljubljana, Večna pot 113, 1000 Ljubljana, Slovenia

[6]Energy Research Institute @ NTU (ERI@N), Research Techno Plaza, X-Frontier Block, Level 5, 50 Nanyang Drive, Singapore

[7]Department of Medical Research, China Medical University Hospital, China Medical University, No. 91 Hsueh-Shih Road, Taichung 40402, Taiwan

[8]Faculty of Electrical Engineering and Computer Science, VSB - Technical University of Ostrava, 17. listopadu 2172/15, 70800 Ostrava, Czech Republic

d.escalera@fz-juelich.de

s.cherevko@fz-juelich.de

Section S1: Physical characterization of cathodic and anodic MoS_x electrodeposits

Physicochemical characterization of the freshly-prepared anodically (a-MoS_{3-x}) and cathodically (c-MoS_2) electrodeposited thin films were performed with SEM and XPS. The SEM characterization of a-MoS_{3-x} and c-MoS_2 is depicted in Figure S1A and B, respectively. Both electrodeposits show a good MoS_x coverage of the glassy carbon substrate, with small grain sizes (<100 nm) comparable to previous reports on electrochemically deposited MoS_x[1, 2]. The XPS survey spectra of a-MoS_{3-x} and c-MoS_2 are shown in Figure S1C and D, and confirm the presence of Mo and S in both deposits along with N from the precursor and O and C arising from both substrate and environment exposure[3]. Further integration of the S and Mo survey spectra yielded S-to-Mo ratios of 2.8 for a-MoS_{3-x} and 1.5 for c-MoS_2. The high-resolution spectra of the Mo 3*d* region for the anodic and cathodic deposit are shown in Figure S1E and Figure S1F, respectively. The deconvoluted peaks at binding energies of 226.4 eV (Figure S1F) and 226.1, 227.4 and 229.0 eV (Figure S1E) were assigned to the S 2*s* peaks in accordance with literature[4, 5]. The deconvolution revealed for both deposits three doublets for Mo with binding energies for the Mo $3d_{5/2}$ signals of 229.3, 230.1 and 231.1 eV for the anodic deposit and 229.1, 230.4 and 232.2 eV for the cathodic deposit. These three doublets were assigned to Mo(IV), Mo(V) and Mo(VI), respectively[5, 6]. The quantification shows in agreement with literature that in both deposits Mo(IV) is the dominant oxidation state with 72 at. % in the anodic, and 50 at. % in the cathodic MoS_x[5, 7]. The presence of Mo(VI) in the deposits points on the presence of MoO_3[5]. The comparable high amount of Mo(VI) in the cathodic deposition gives a possible explanation for the low S-to-Mo ratio found from the survey spectrum. The recalculation of the S-to-Mo ratio results in a value of 1.9. A possible explanation for the presence Mo(V) could be the formation of intermediate products[7, 8].

The high-resolution spectra of the S 2*p* region for the anodic deposition is depicted in Figure S1G for the cathodic deposition in Figure S1H. For the deconvolution of S 2*p* region in the anodic deposit, three doublets with the binding energies of the S $2p_{1/3}$, and S $2p_{1/2}$ signals of 163.5, 164.7 eV and 163.2, 164.4 eV and 161.9 eV, 163.1 eV were respectively attributed to S(0), S^{2-} and S_2^{2-} species[9]. For the cathodic deposit, two doublets with the binding energies of the S $2p_{1/3}$, S $2p_{1/2}$ signals were located at 163.1, 164.3 eV and at 161.8 eV, 162.9 eV were assigned to S^{2-} and S_2^{2-} species. The exact quantification of the S(0), S^{2-} and S_2^{2-} is not possible because of overlapping signals of apical, terminal and bridging S^{2-} and S_2^{2-}species[7]. Nevertheless, the appearance and shape of the high-resolution XPS spectra for Mo 3*d* and S 2*p* are consistent to previously reported spectra measured in MoS_3 and MoS_2[10, 11]. Therefore, the SEM and XPS investigations confirm the electrochemical formation of MoS_2 and MoS_{3-x}.

Figure S1. Physicochemical characterization of MoS_x allotropes

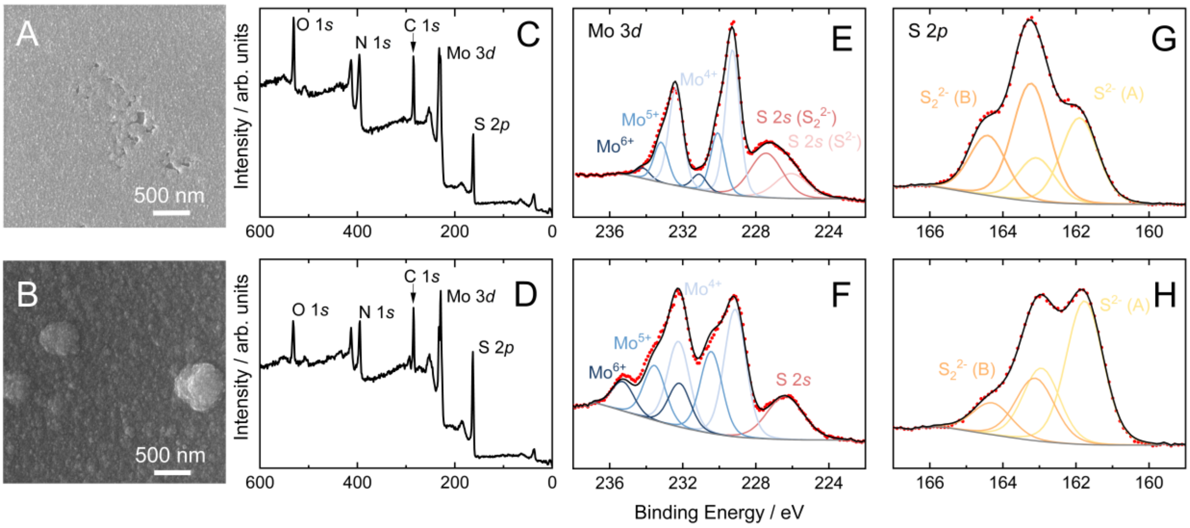

Physicochemical characterization of the anodically (A, C, E, G) and cathodically (B, D, F, H) electrodeposited MoS_x. A, B) Show the scanning electron micrographs, C, D) the XPS survey spectra, E, F) the XPS spectra of the Mo 3*d* region, and G, H) the XPS spectra of the S 2*p* region.

Figure S2. Loading-normalized Mo and S dissolution at varying LPLs

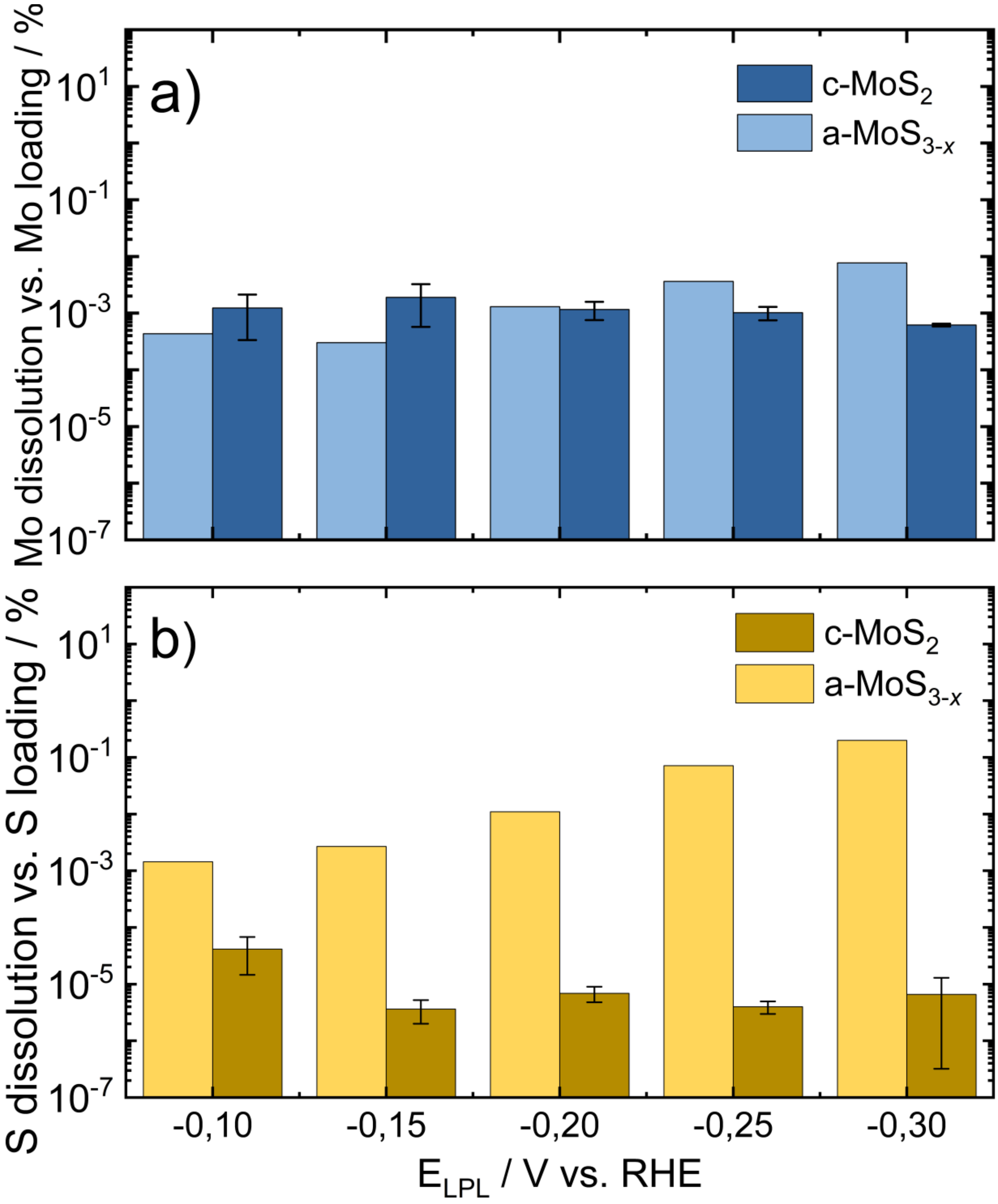

Graphical representation of the loading-normalized integrated dissolution of a) Mo (blue) and b) S (yellow) as a function of the LPL. For electrochemical protocol, see Figure 1. Scan rate: 5 mV s^{-1}.

Figure S3. Mo and S dissolution onsets at varying LPLs

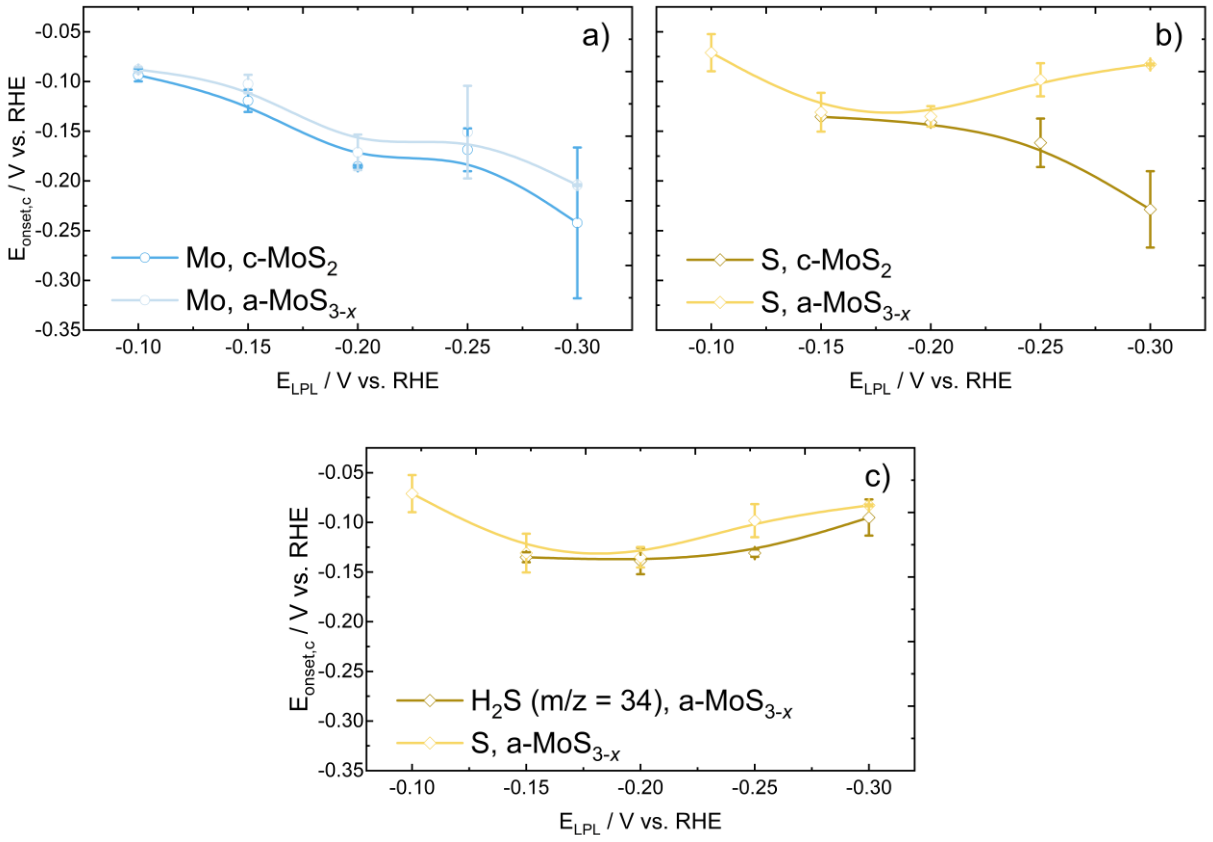

Top: Graphical representation of dissolution onset potentials of a) Mo (blue) and b) S (yellow) as a function of the LPL. c) Side-by-side comparison of S dissolution onsets from ICP-MS and EC-MS data. Scan rate: 5 mV s^{-1}.

Figure S4. Mo and S dissolution at varying LPLs for [Mo_3S_{13}]-based catalysts

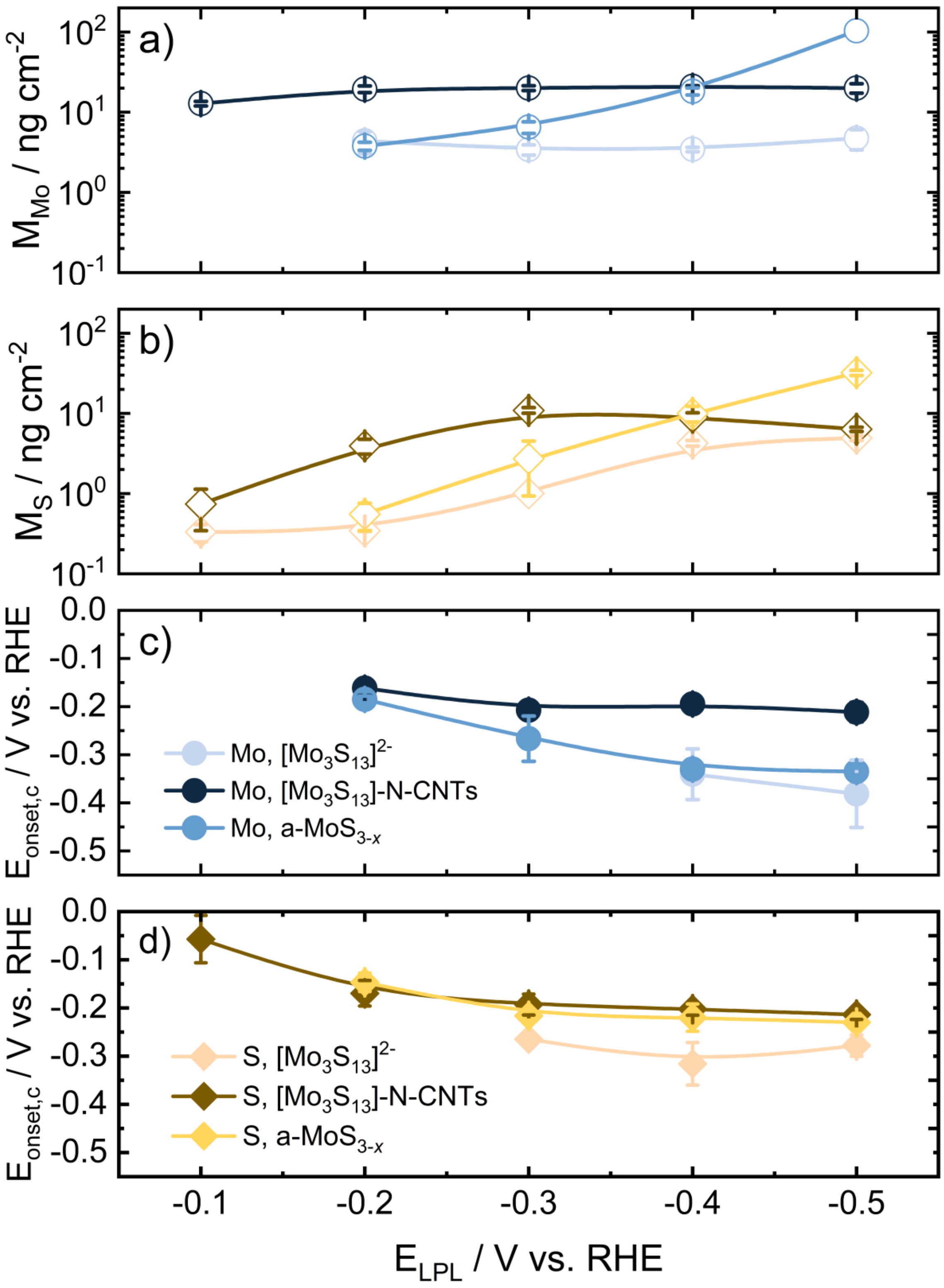

Total integrated dissolution of a) Mo (blue) and b) S (yellow) and the corresponding experimental dissolution onset potentials of c) Mo and d) S as a function of the LPL. CVs recorded from 0 V_{RHE} to LPLs in the range $-0.1 \leq E_{UPL} \leq -0.5$ V. Scan rate: 5 mV s^{-1}.

Figure S5. Loading-normalized Mo and S dissolution at varying LPLs for [Mo_3S_{13}]-based catalysts

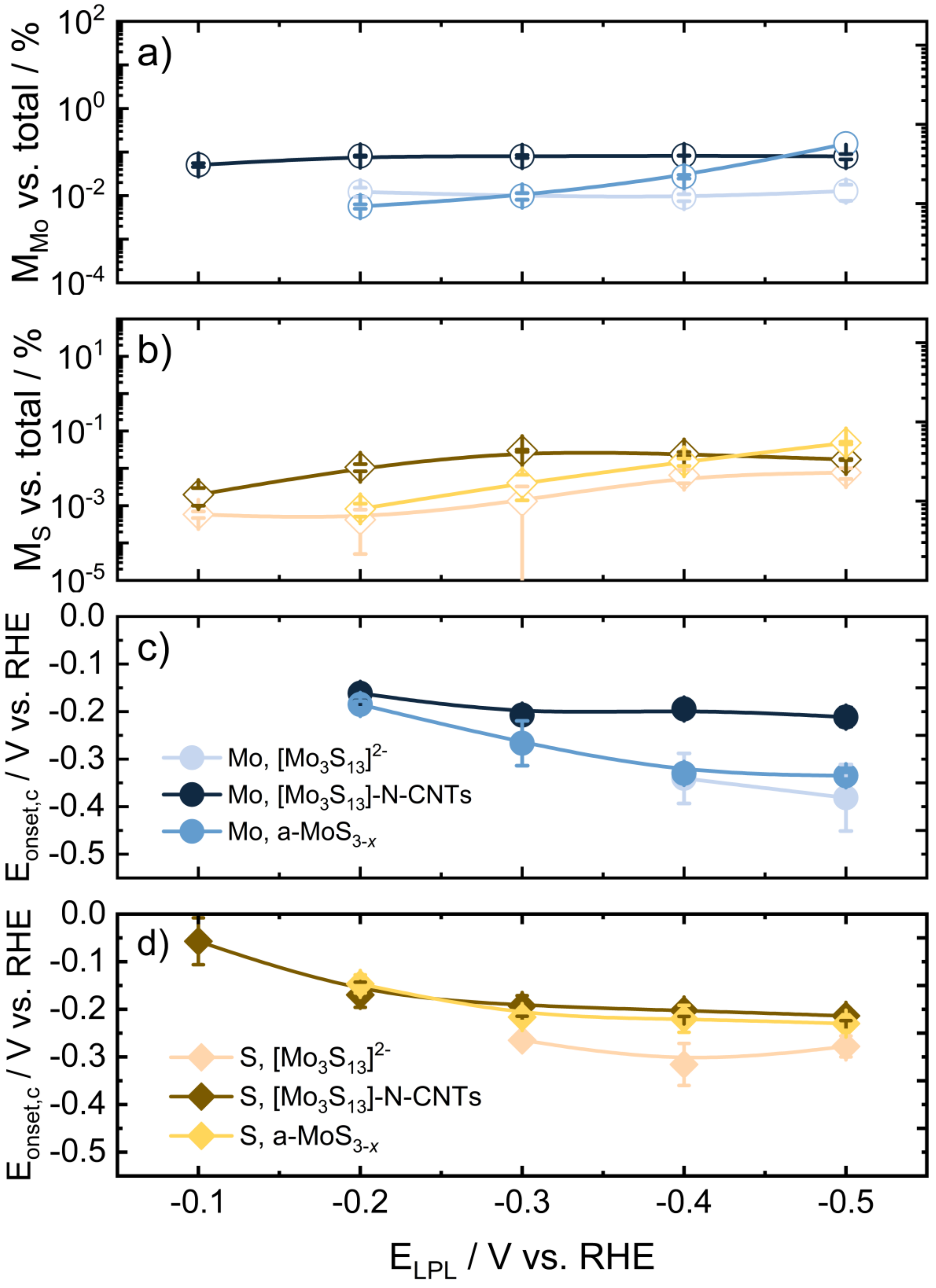

Loading-normalized integrated dissolution of a) Mo (blue) and b) S (yellow) and the corresponding experimental dissolution onset potentials of c) Mo and d) S as a function of the LPL. CVs recorded from 0 V_{RHE} to LPLs in the range $-0.1 \leq E_{UPL} \leq -0.5$ V. Scan rate: 5 mV s^{-1}.

Figure S6. Integrated Mo and S dissolution during HER start-up/shutdown

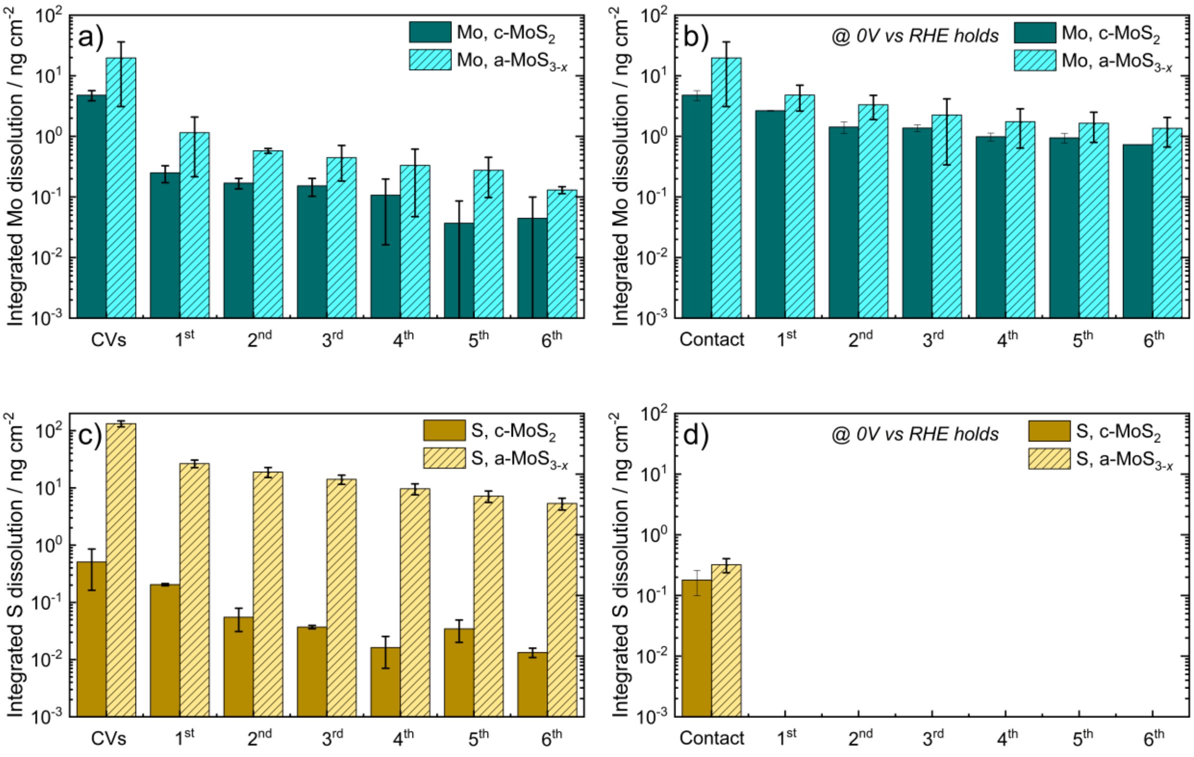

Total dissolution data for a-b) Mo and c-d) S integrated at each consecutive -1 mA cm^{-2}_{geom} HER holds (left, "start-up" cycle) and 0 V_{RHE} (right, "shutdown" cycle). Labels: a-MoS_{3-x} (light blue/yellow) and c-MoS_2 (dark blue/yellow).

Figure S7. Loading-normalized integrated Mo and S dissolution during HER start-up/shutdown

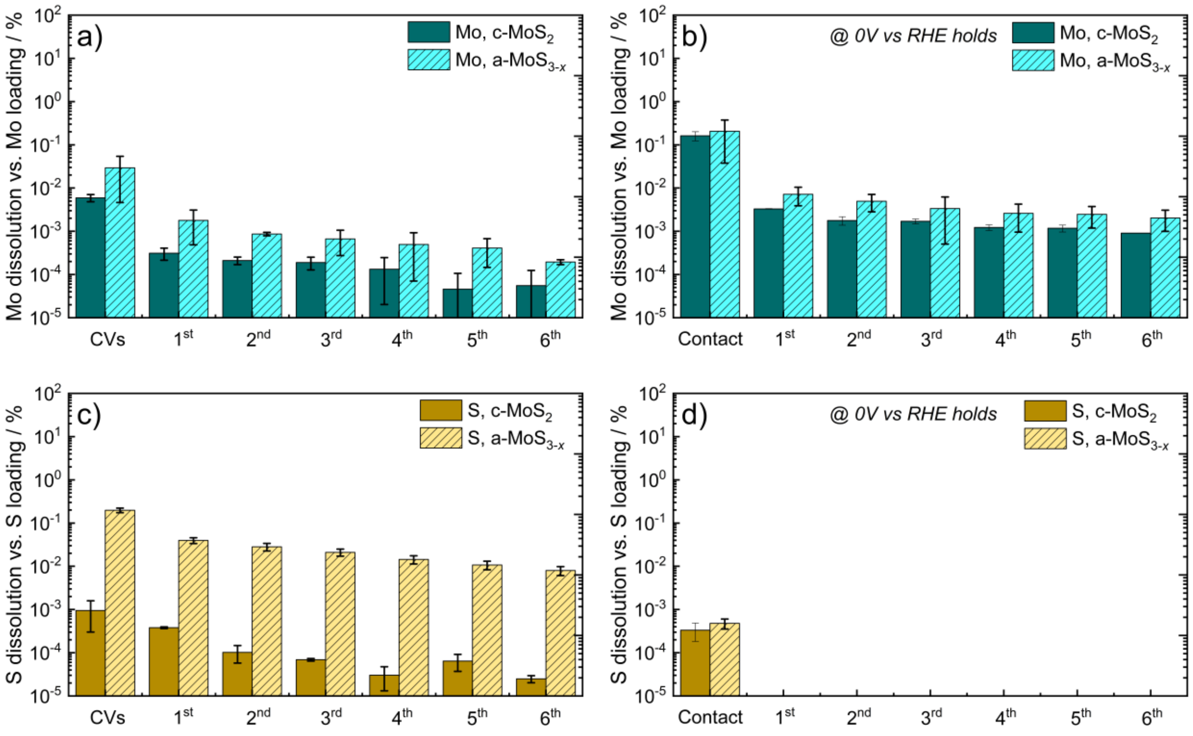

Loading-normalized dissolution data for a-b) Mo and c-d) S integrated at each consecutive -1 mA cm^{-2}_{geom} HER holds (left, “start-up” cycle) and 0 V_{RHE} (right, “shutdown” cycle). Labels: a-MoS_{3-x} (light blue/yellow) and c-MoS_2 (dark blue/yellow).

Figure S8. Compiled Mo S-number (e^-) and electrochemical potentials during HER start-up/shutdown

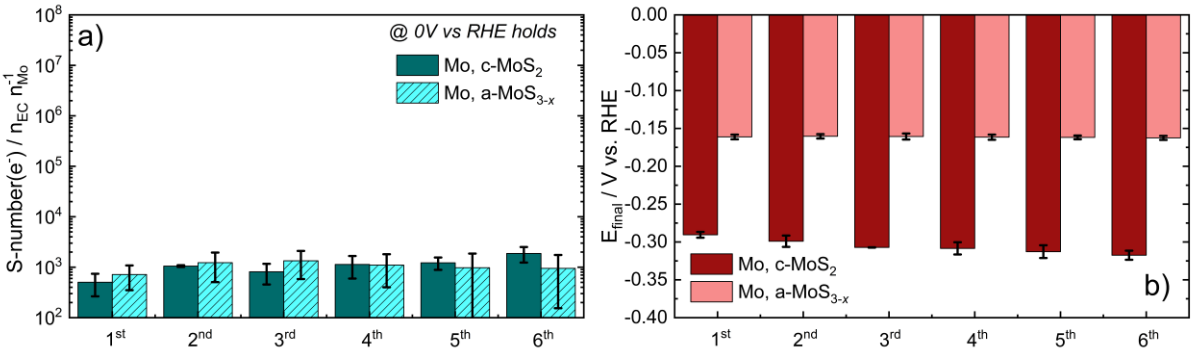

a) Compilation of S-numbers(e^-) obtained during 0V vs. RHE holds for and b) hold-dependent HER activity (right).

Figure S9. Online ICP-MS data during HER start-up/shutdown for [Mo_3S_{13}]-based catalysts

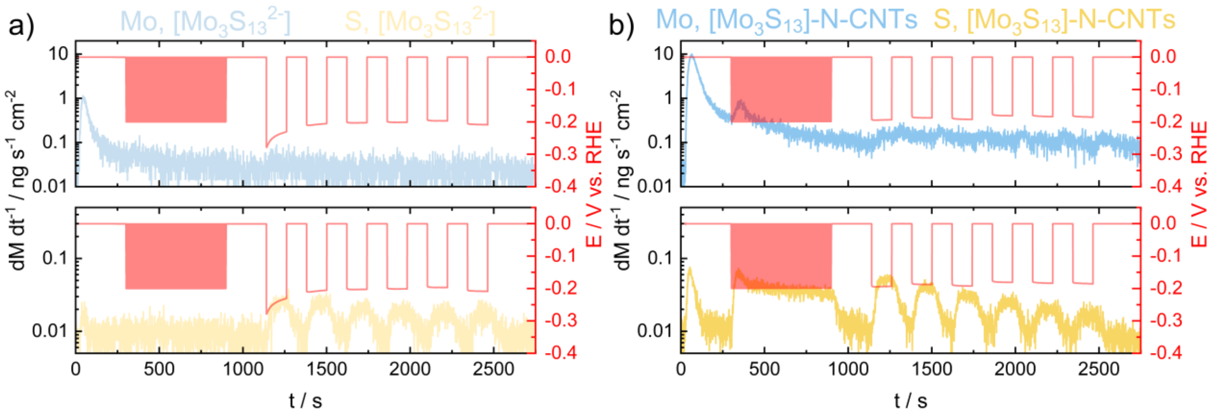

Online ICP-MS dissolution data obtained for Mo (blue) and S (yellow) in a) pristine and b) N-CNT anchored [Mo_3S_{13}]$^{2-}$ catalyst during start-up/shut-down stress tests. Preconditioning: 150 CVs, 0 to -0.20 V_{RHE}, 100 mV s^{-1}.

Figure S10. Compiled stability data for $[Mo_3S_{13}]$-based catalysts during HER start-up/shutdown

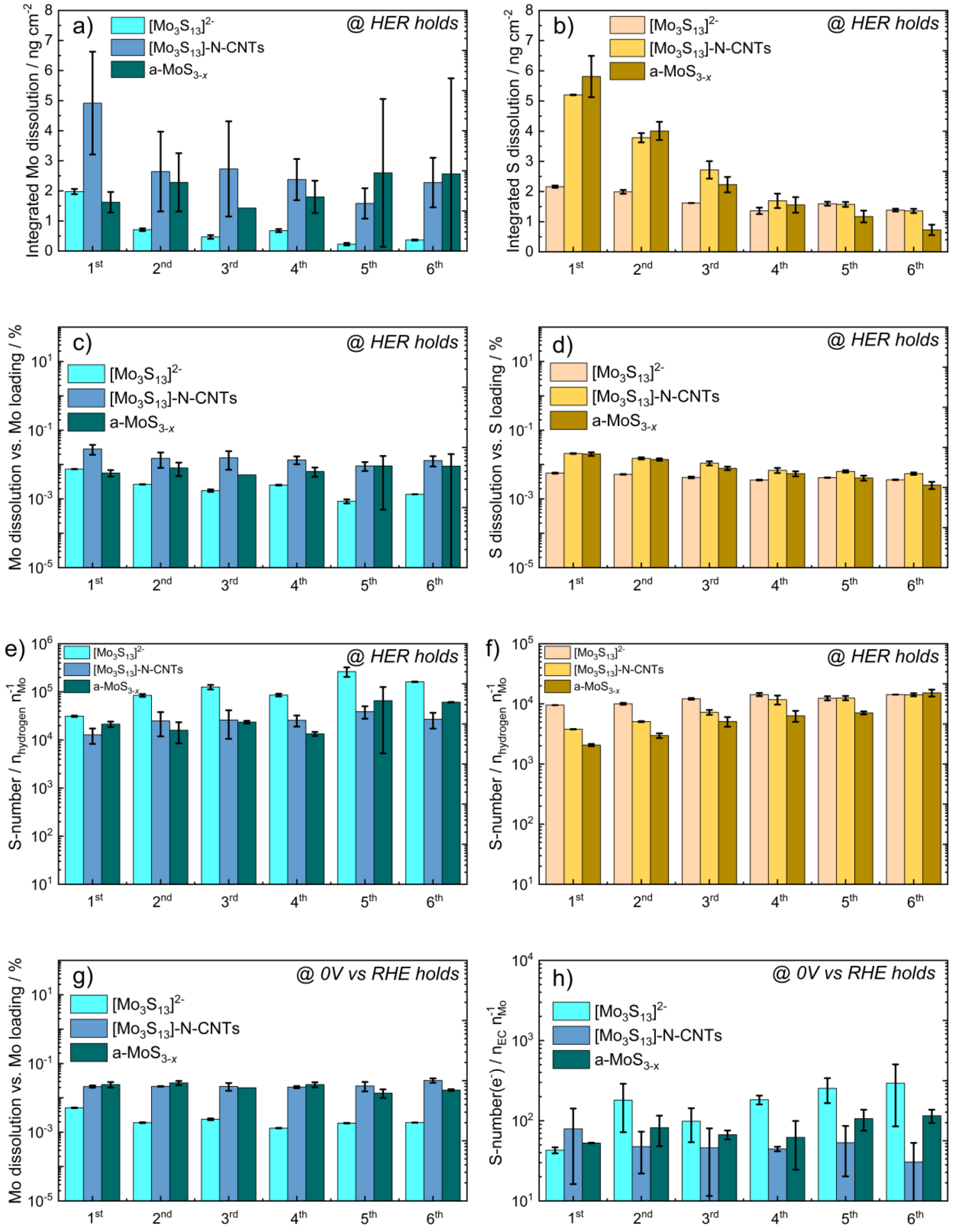

a-b) Total and c-d) loading-normalized dissolution data for Mo (a,c) and S (b,d) integrated at each consecutive -1 mA cm^{-2}_{geom} HER holds. e-f) Compilation of S-numbers obtained for a-

MoS_{3-x}, $[Mo_3S_{13}]^{2-}$ and $[Mo_3S_{13}]$-N-CNTs during start-up/shut-down stress tests for Mo (left) and S (right) under HER conditions. g-h) Loading-normalized Mo dissolution and corresponding S-numbers(e^-) during 0V vs. RHE holds.

Section S2. Impact of anodic potential in MoS_x stability

Voltage reversal from HER operation has been demonstrated here to severely compromise MoS_x stability regardless of the phase. If PEMWEs were to be coupled with intermittent renewable energies that can lead to shutdowns with loss of potential control, it is paramount to evaluate the effect of anodic potentials in the stability of MoS_x, as no dedicated literature is available. To do so, the stability of MoS_x-based catalysts under anodic potentials was evaluated by recording sequential CVs on the pristine MoS_x catalysts from 0 V_{RHE} to UPLs in the range 0.7 – 1.5 V, where each successive cycle incremented its UPL by 200 mV.

When directly comparing a-MoS_{3-x} and c-MoS_2 electrodeposited thin films there are stark differences, observed once both total Mo and S dissolution and their corresponding dissolution onset potentials were plotted vs. the UPLs. First, the integrated Mo dissolution in a-MoS_{3-x} is 10 times higher than c-MoS_2 at any given UPL (Figure S11a-b). Second, S dissolution can only be clearly observed in a-MoS_{3-x}, at potentials beyond 1.1 V_{RHE} and is ca. 3 orders of magnitude lower than that of Mo.

Dissolution onset potentials were additionally estimated for Mo and S (Figure S11c). Interestingly, Mo dissolution onsets for pristine a-MoS_{3-x} and c-MoS_2 were similar (ca. 0.3 V_{RHE}) but after successive electro-oxidative cycles these shifted to higher values for c-MoS_2, in contrast with a-MoS_{3-x} where they peaked at UPL= 1.3 V_{RHE} (Mo onset ~0.9 V_{RHE}). Below this UPL value, Mo onsets for a-MoS_{3-x} are 150-200 mV lower than c-MoS_2. S dissolution could only be found for a-MoS_{3-x} at E >1.1 V_{RHE}. The high potentials recorded during anodic S dissolution ($1.2 \leq E \leq 2.05$ V_{RHE}) resemble the results obtained on S-coordinated single-atom Pt electrocatalysts, where a S onset dissolution potential of >1.1 V_{RHE} was reported[12]. We could conclude that, as previously suggested, electro-oxidation pathways of a-MoS_{3-x} and c-MoS_2 present intrinsic differences. While c-MoS_2 is more prone to oxidize (i.e. earlier dissolution onsets), its overall dissolution at anodic potentials is lower compared to a-MoS_{3-x}, indicating a degree of surface passivation confirmed by the upward shift in Mo dissolution onsets. It is noteworthy to mention that a-MoS_{3-x} is further destabilized once S loss is detected, indicating the formation of unstable undercoordinated Mo_xS_y moieties beyond a specific UPL.

Figure S11. Total, loading-normalized integrated Mo and S dissolution and corresponding dissolution onsets at varying UPLs

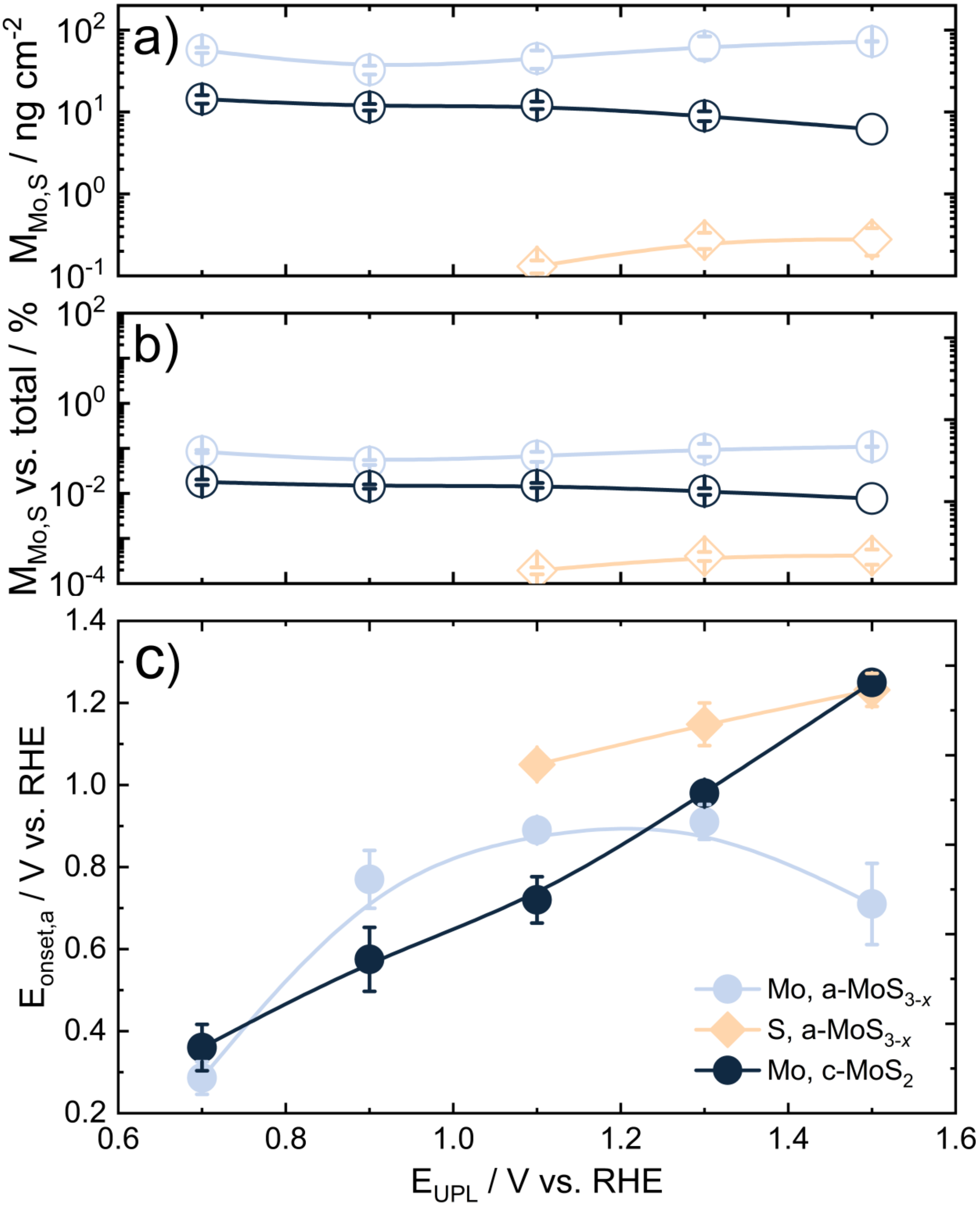

a) Total and b) loading-normalized integrated dissolution of Mo (blue) and S (yellow) and the corresponding experimental dissolution onset potentials for c) a-MoS_{3-x} and c-MoS_2 and as a function of the UPL. Scan rate: 5 mV s^{-1}.

Analogous experiments performed on [Mo_3S_{13}]-based catalysts at equivalent loadings (ca. 60 μg_{cat} cm^{-2}) are compiled in Figures S12 and S13. Despite having an identical motif, [Mo_3S_{13}]-based catalysts presented different dissolved amounts and dissolution onsets of Mo per electro-oxidative cycle. The earliest Mo onset (0.37± 0.03 V_{RHE}) and highest Mo dissolution (97±16 ng cm^{-2}) were found for MoS_x-N-CNT, and can be ascribed to higher Mo_3S_{13} catalyst utilization and improved electron transfer properties by the highly-conducting, percolated N-CNT support. In line with our previous findings, S dissolution was not observed at the selected UPLs (Figure S12) but was present instead when performing anodic galvanostatic holds (Figure S14). Thus, the more compact yet poorly percolated network in a-MoS_{3-x} would be less prone to be oxidized than the highly-dispersed Mo_3S_{13} clusters in highly conducting N-CNTs.

Figure S12. Online ICP-MS data at varying UPLs for [Mo_3S_{13}]-based catalysts

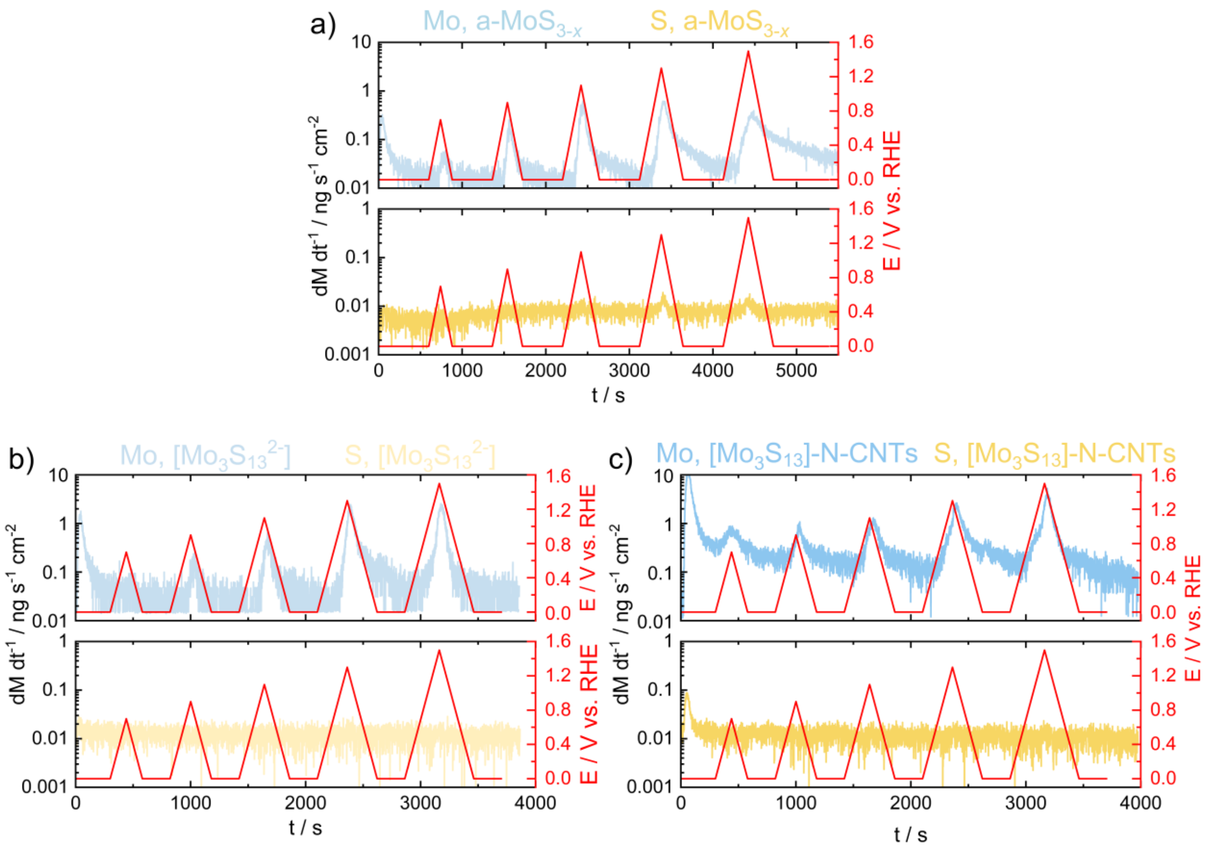

Online ICP-MS dissolution data obtained for successive linear sweep voltammograms (LSVs) with varying upper potential limit (UPL) values for Mo (blue) and S (yellow). LSVs recorded from 0 V_{RHE} to UPLs in the range $0.7 \leq E_{UPL} \leq 1.5$. Labels: a) a-$MoS_{3-x}$, b) [$Mo_3S_{13}$]$^{2-}$, c) [$Mo_3S_{13}$]-N-CNTs. Scan rate: 5 mV s^{-1}.

Figure S13. Total, loading-normalized integrated Mo and S dissolution and corresponding dissolution onsets at varying UPLs for [Mo_3S_{13}]-based catalysts

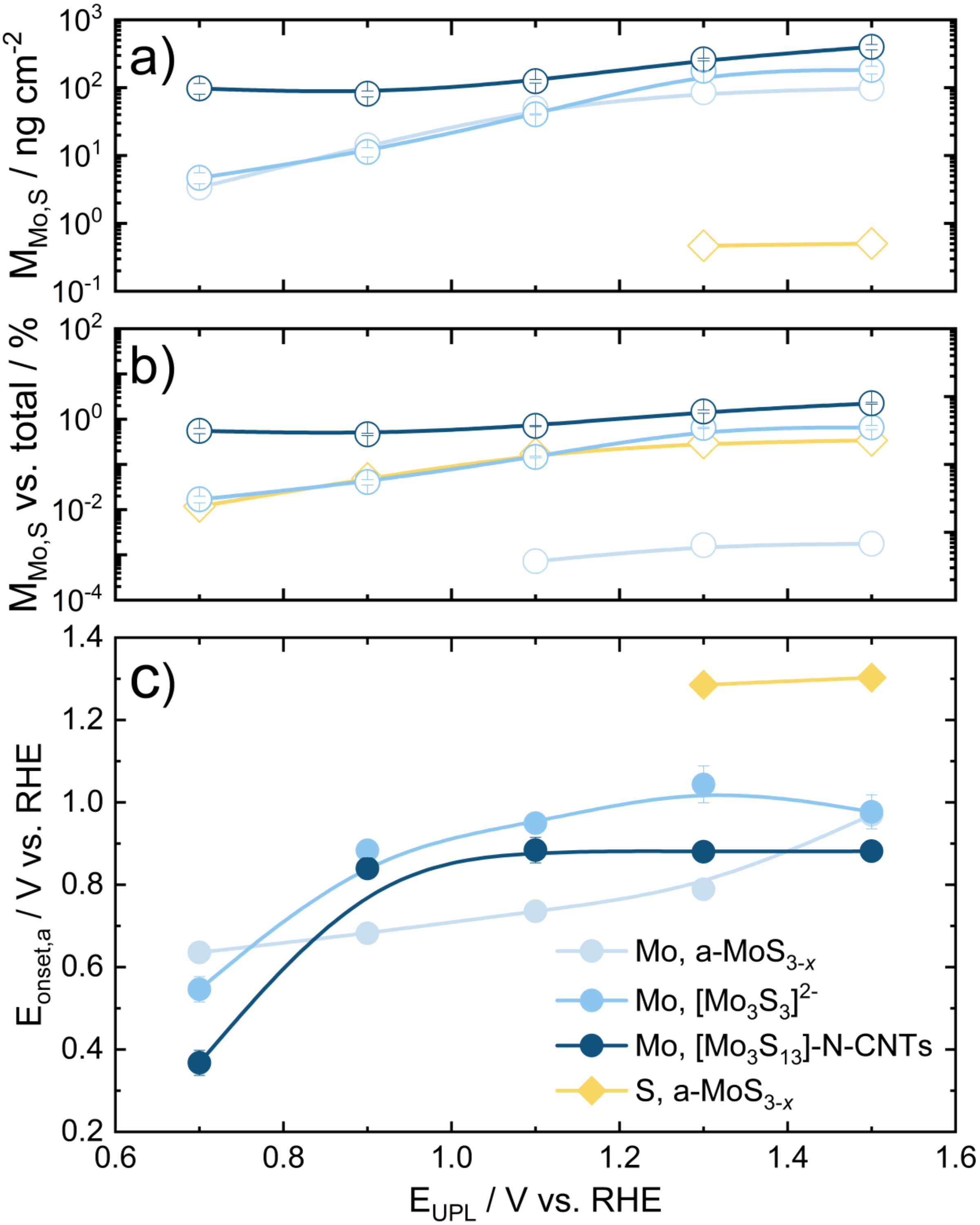

a) Total and b) loading-normalized integrated dissolution of Mo (blue) and S (yellow), and c) experimental dissolution onset potentials for the [Mo_3S_{13}]-based catalysts (a-MoS_{3-x}, [Mo_3S_{13}]^{2} and MoS_x-N-CNT) as a function of the UPL. Scan rate: 5 mV s^{-1}.

Compared with the theoretical oxidation potential reported from both the Mo-S-O-H Pourbaix diagram (ca. 0.27 V_{RHE})[13] and the recently SCAN-calculated Pourbaix decomposition diagram (0.35 V_{RHE})[14] for crystalline MoS_2, there are minor discrepancies with the Mo onsets reported here. For all MoS_x materials studied, these should arise from the higher presence of defects (i.e. $Mo^{5+/6+}$ oxides) and the intrinsic structural differences compared to crystalline MoS_2, besides the different concentrations of dissolved [Mo^{x+}] species used to calculate the thermodynamic equilibrium potentials (ca. 10^{-8} and 10^{-6} M, respectively). However, the positively shifted Mo onsets upon repetitive oxidative cycling indicate a stabilization of the Mo centers which cannot be explained by the previous hypotheses alone.

To gather further insights on the surface oxidation states before/after electro-oxidative cycling, ex-situ XPS measurements were performed on the $[Mo_3S_{13}]^{2-}$ and MoS_x-N-CNT catalysts (Figures S15 and S16, respectively)[15]. As foreseen, an increased presence of oxidized $Mo^{5+}O_xS_y/Mo^{6+}$ surface species was observed: 11 at. % vs. 4.5 % in pristine $[Mo_3S_{13}]^{2-}$; 17 at. % vs. 3.9 % in pristine MoS_x-N-CNT. Additionally, the relative contribution of the S_2^{2-}/S^{2-} species in the S 2*p* spectra was modified. The lower binding energy spin-orbit doublet $2p_{3/2:1/2}$ ascribed to terminal S_2^{2-} ($S_2^{2-}{}_{term}$) and/or unsaturated S^{2-} ($S^{2-}{}_{unsat}$) is less prominent after electro-oxidation than the high-binding energy doublet related to bridging S_2^{2-} ($S_2^{2-}{}_{bridg}$) and apical S^{2-} ($S^{2-}{}_{ap}$). The relative loss of the $S_2^{2-}{}_{term}/S^{2-}{}_{unsat}$ doublet (expressed as a ratio $S_2^{2-}{}_{term}/S^{2-}{}_{unsat}$: $S_2^{2-}{}_{bridg}$/ $S^{2-}{}_{ap}$, after testing vs. pristine) is 40:60 vs. 49:51 at. % for $[Mo_3S_{13}]^{2-}$ and 41:59 vs. 49:51 at. % for MoS_x-N-CNT. Such preferential oxidation of the weakly-bound, more readily-oxidized low binding S moieties was previously found for a-MoS_{3-x} materials after partial anodic stripping[9] and ascribed to $S_2^{2-}{}_{term}$ loss[16].

Galvanostatic holds at oxidative potentials (+1 mA cm^{-2}, 10 mins) further corroborated that the increased $Mo^{5+}O_xS_y/Mo^{6+}$ surface contents and $S_2^{2-}{}_{term}/S^{2-}{}_{unsat}$ loss are linked to the harshness of the electro-oxidative protocol (Figure S15-16). Thus, we believe that the stabilization of $[Mo_3S_{13}]$-based catalysts under anodic potentials lies in the selective conversion of $S_2^{2-}{}_{term}$ to S_2^{-}/SO_x^{y-} via a 1 H^+: 2e^- proton-coupled electron transfer mechanism as proposed for pristine a-MoS_{3-x}[17] and crystalline MoS_2[18]. Beyond the 0.3 V_{RHE} Mo onset potential, the readily oxidized $S_2^{2-}{}_{term}$ would yield undercoordinated Mo sites, which can then electro-oxidize by forming Mo-O or Mo=O bonds but would be ultimately limited by the depletion of $S_2^{2-}{}_{term}$ sites. Beyond a certain potential threshold, proposed here to be > 1.1 V_{RHE}, the remaining S groups would electro-oxidize facilitating the further oxidation of Mo sites to MoO_2/MoO_3 and ultimately $HMoO_4^-$. For c-MoS_2, the earlier electro-oxidation could arise from the higher relative contents

of the oxidation-prone $S_2^{2-}{}_{term}$ ligands in the structure, closer to that of crystalline MoS_2 which should oxidize at earlier potentials.

Figure S14. Online ICP-MS data at varying UPLs for [Mo_3S_{13}]-based catalysts

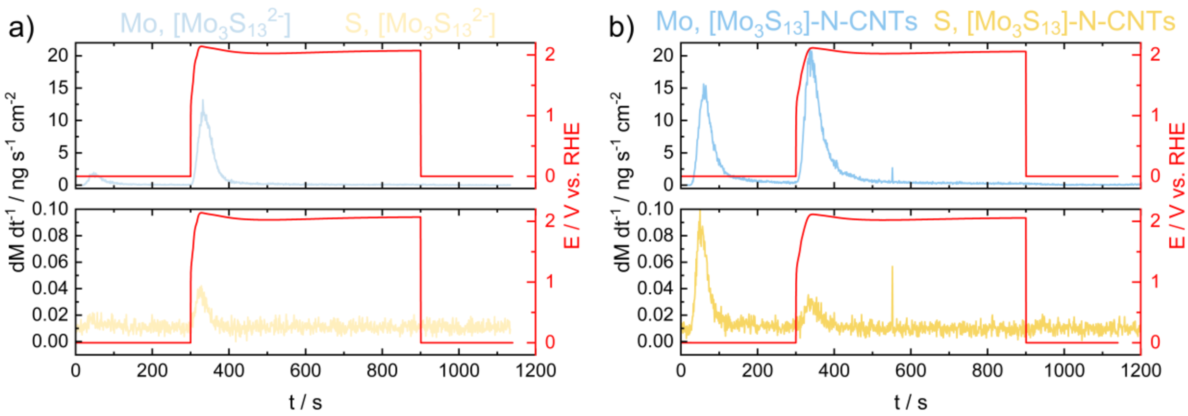

Online ICP-MS dissolution data recorded for Mo (blue, upper pane) and S (yellow, lower pane) under a galvanostatic anodic hold (+1 mA $cm^{-2}{}_{geom}$, 10 mins) exerted to a) pristine and b) N-CNT anchored $[Mo_3S_{13}]^{2-}$ catalyst.

Figure S15. Ex-situ XPS data on pristine $[Mo_3S_{13}]^{2-}$ before/after electro-oxidative experiments

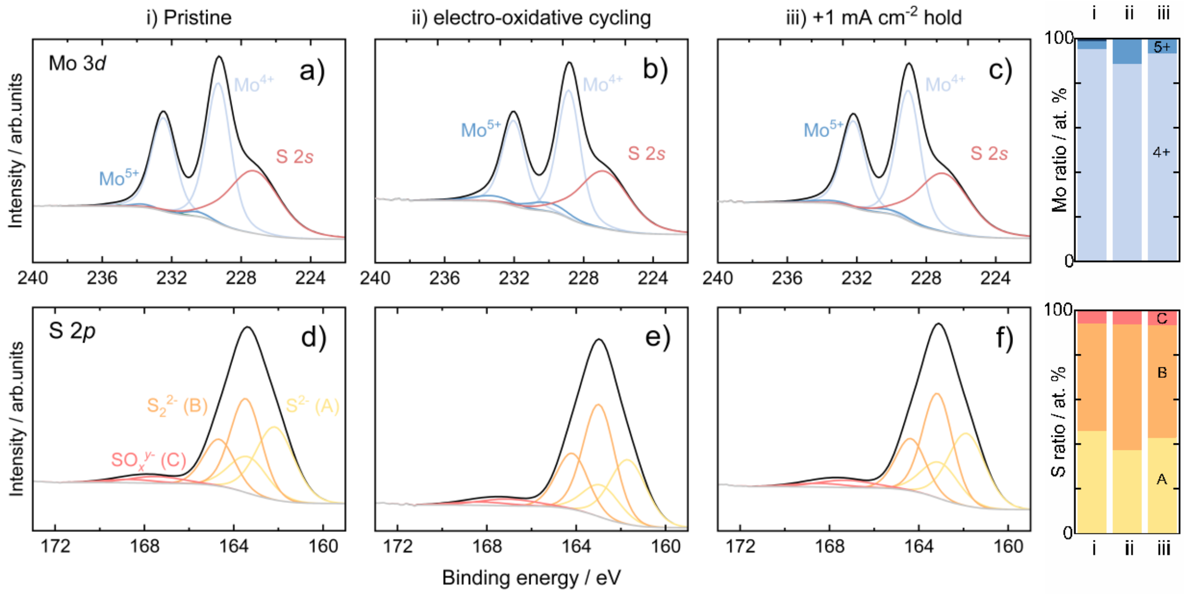

High-resolution Mo 3*d* (a-c) and S 2*p* (b-e) XPS spectra of pristine $[Mo_3S_{13}]^{2-}$ before electrochemistry (I; a,d), after electro-oxidative cycling (II; b,e), and after a +1 mA cm^{-2} galvanostatic hold (III; c,f). Labels: cumulative peak fit (black), Mo^{4+} $3d_{5/2:3/2}$ (light blue), $Mo^aO_bS_c$ $3d_{5/2:3/2}$ (blue), Mo^{6+} $3d_{5/2:3/2}$ (dark blue), S $2p_{3/2:1/2}$ (S^{2-}, yellow), S $2p_{3/2:1/2}$ (S_2^{2-}, orange) and S $2p_{3/2:1/2}$ (SO_x^{y-}, red).

Figure S16. Ex-situ XPS data on Mo_3S_{13}-NCNT before/after electro-oxidative experiments

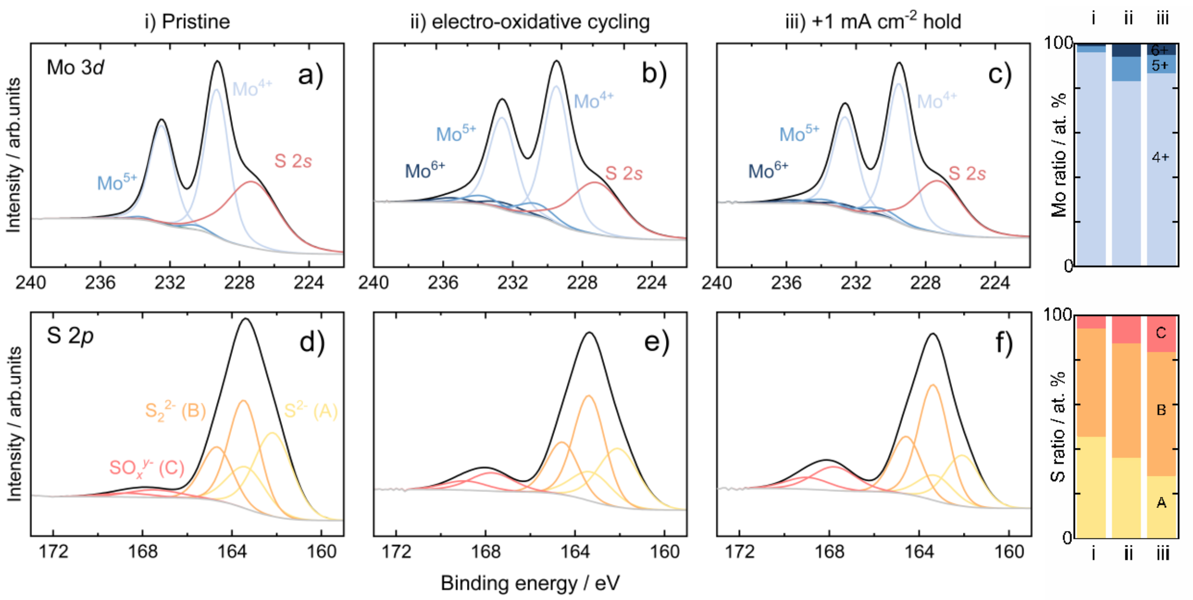

High-resolution Mo 3*d* (a-c) and S 2*p* (d-f) XPS spectra of Mo_3S_{13}-NCNT before electrochemistry (I; a,d), after electro-oxidative cycling (II; b,e), and after a +1 mA cm^{-2} galvanostatic hold (III; c,f). Labels: cumulative peak fit (black), Mo^{4+} $3d_{5/2:3/2}$ (light blue), $Mo^{a}O_{b}S_{c}$ $3d_{5/2:3/2}$ (blue), Mo^{6+} $3d_{5/2:3/2}$ (dark blue), S $2p_{3/2:1/2}$ (S^{2-}, yellow), S $2p_{3/2:1/2}$ (S_2^{2-}, orange) and S $2p_{3/2:1/2}$ (SO_x^{y-}, red).

Section S3: Hydrogen evolution and dissolution pathways under HER potentials

The redox processes responsible for the electrocatalytic activity in c-MoS_2 and a-MoS_{3-x}, based upon the hypothesis of the Mo-H active site universality postulated by Bau et al.,[19] along with the dissolution pathways proposed in this work are as follows:

S3.1 Anodically-electrodeposited MoS_x thin films (a-MoS_{3-x})

For ease of convenience, Scheme 2 does not include the electrochemical loss of apical S^{2-} (S^{2-}_{ap}) moieties, shown as follows

$$Mo^{4+}\left[(S)_{ap}\right]^{2-} + 1\,H_3O^{+} + 1e^{-} \rightarrow\; Mo^{4+} - \blacksquare + 1\,H_2O + 1\,HS^{-}$$

This process can take place alongside with the terminal S_2^{2-} ($S_2^{2-}{}_{term}$) loss, which is postulated to be responsible for the appearance of the Mo^{3+} hydride sites and ultimately the HER activity

$$Mo^{4+}\ [(S-S)_{term}]^{2-} + 2\ H_3O^+ + 2e^- \rightarrow [S^{2-} - Mo^{4+} - \blacksquare] + 2\ H_2O + 1\ H_2S \qquad (I)$$

$$[S^{2-} - Mo^{4+} - \blacksquare] + 1\ H_3O^+ + 2e^- \rightarrow\ [S^{2-} - Mo^{3+} - H]\ \ + 1\ H_2O \qquad (II)$$

$$[S^{2-} - Mo^{3+} - H]\ + 1\ H_3O^+ \rightarrow\ [S^{2-} - Mo^{4+} - \blacksquare]\ + 1\ H_2O\ \ + 1\ H_2 \qquad (III)$$

Alternatively, based on previous work by Tran et al.,[20] a Mo^{5+} hydride pathway could also be responsible for the HER electrocatalytic activity

$$[S^{2-} - Mo^{3+} - H]\ + 2\ H_3O^+ \rightarrow\ [\blacksquare - Mo^{3+} - H]\ \ + 2\ H_2O + 1\ H_2S \qquad (IV)$$

$$[\blacksquare - Mo^{3+} - H]\ \ + 1\ H_3O^+\ \ \rightarrow\ \ [\blacksquare - Mo^{5+} - \blacksquare] + 1\ H_2O + 1\ H_2 \qquad (V)$$

$$[\blacksquare - Mo^{5+} - \blacksquare]\ \ + 1\ H_3O^+\ + 2e^- \rightarrow\ [\blacksquare - Mo^{3+} - H]\ \ + 1\ H_2O \qquad (VI)$$

Finally, two redox processes are proposed to be responsible for the observed Mo dissolution at cathodic potentials

$$Mo^{4+}\left[(S-S)_{term/br}\right]^{2-} - [\blacksquare - Mo^{3+} - H]\ \ + 2\ H_3O^+\ + 2e^-$$
$$\rightarrow\ HS -\ Mo^{4+} - SH +\ [Mo^{3+} - H]^{2+}{}_{aq}\ \ + 2\ H_2O \qquad (VII)$$

Or

$$Mo^{4+}\left[(S-S)_{term/br}\right]^{2-} - [S^{2-} - Mo^{3+} - H]\ + 2\ H_3O^+ + 2e^-$$
$$\rightarrow\ HS -\ Mo^{4+} - SH +\ [S^{2-} - Mo^{3+} - H]_{aq}\ \ + 2\ H_2O \qquad (VIII)$$

S3.2 Cathodically-electrodeposited MoSx thin films (c-MoS2)

An analogous HER mechanism can be proposed for c-MoS_2, triggered in this case by unsaturated S^{2-} (S^{2-}_{unsat}) loss

$$S^{2-} - Mo^{4+} - S^{2-} + 2\,H_3O^+ + 2e^- \rightarrow\ [S^{2-} - Mo^{4+} - \blacksquare] + 2\,H_2O + 1\,H_2S \qquad (I')$$

$$[S^{2-} - Mo^{4+} - \blacksquare] + 1\,H_3O^+ + 2e^- \rightarrow\ [S^{2-} - Mo^{3+} - H]\ \ + 1\,H_2O \qquad (II')$$

$$[S^{2-} - Mo^{3+} - H] + 1\,H_3O^+ \rightarrow\ [S^{2-} - Mo^{4+} - \blacksquare]\ \ + 1\,H_2 \qquad (III')$$

Finally, two redox processes are proposed to be responsible for the observed Mo dissolution at cathodic potentials

$$[S^{2-} - Mo^{3+} - H]\ + 2\,H_3O^+ \rightarrow\ [\blacksquare - Mo^{3+} - H]\ \ + 2\,H_2O + 1\,H_2S \qquad (IV')$$

$$S^{2-} - Mo^{4+} - S^{2-} - [\blacksquare - Mo^{3+} - H]\ \ + 2\,H_3O^+ + 2e^-$$
$$\rightarrow\ HS - \ Mo^{4+} - SH + \ [Mo^{3+} - H]^{2+}{}_{aq}\ \ +\ H_2O \qquad (V')$$

Or

$$S^{2-} - Mo^{4+} - S^{2-} - [S^{2-} - Mo^{3+} - H]\ + 2\,H_3O^+ + 2e^-$$
$$\rightarrow\ HS - \ Mo^{4+} - SH + \ [S^{2-} - Mo^{3+} - H]_{aq}\ \ + 2\,H_2O \qquad (VI')$$

Figure S17. Online ICP-MS data during sequential galvanostatic holds

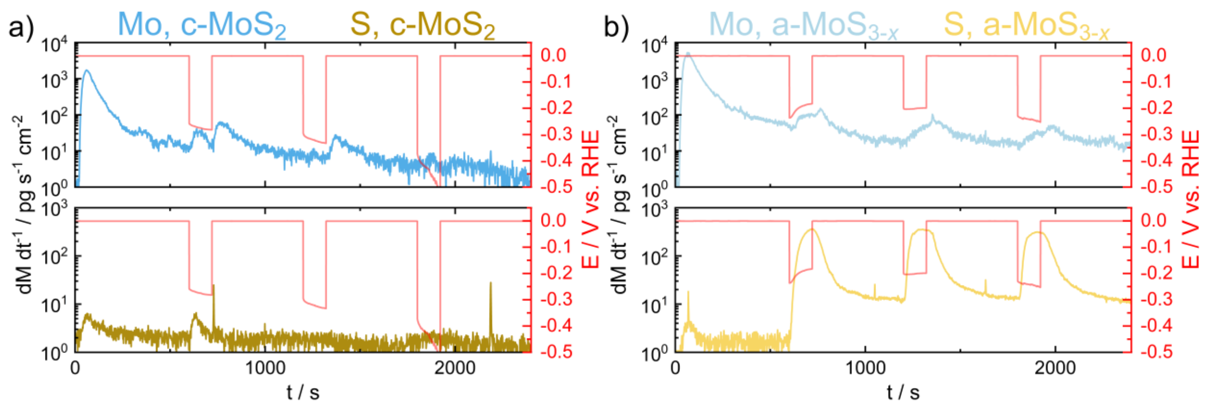

Online ICP-MS dissolution data obtained for a) c-MoS_2 and b) a-$MoS_{3\text{-}x}$ during sequential galvanostatic holds with increasing cathodic geometric current density (j_{geom}= -1, -2, -5 mA cm^{-2}, 2 min) values for Mo (blue) and S (yellow). Potentiostatic holds at 0 V_{RHE} (8 mins) are employed here to resolve dissolution signals at 0 V vs. RHE.

Figure S18. Compiled S-numbers obtained during sequential galvanostatic holds

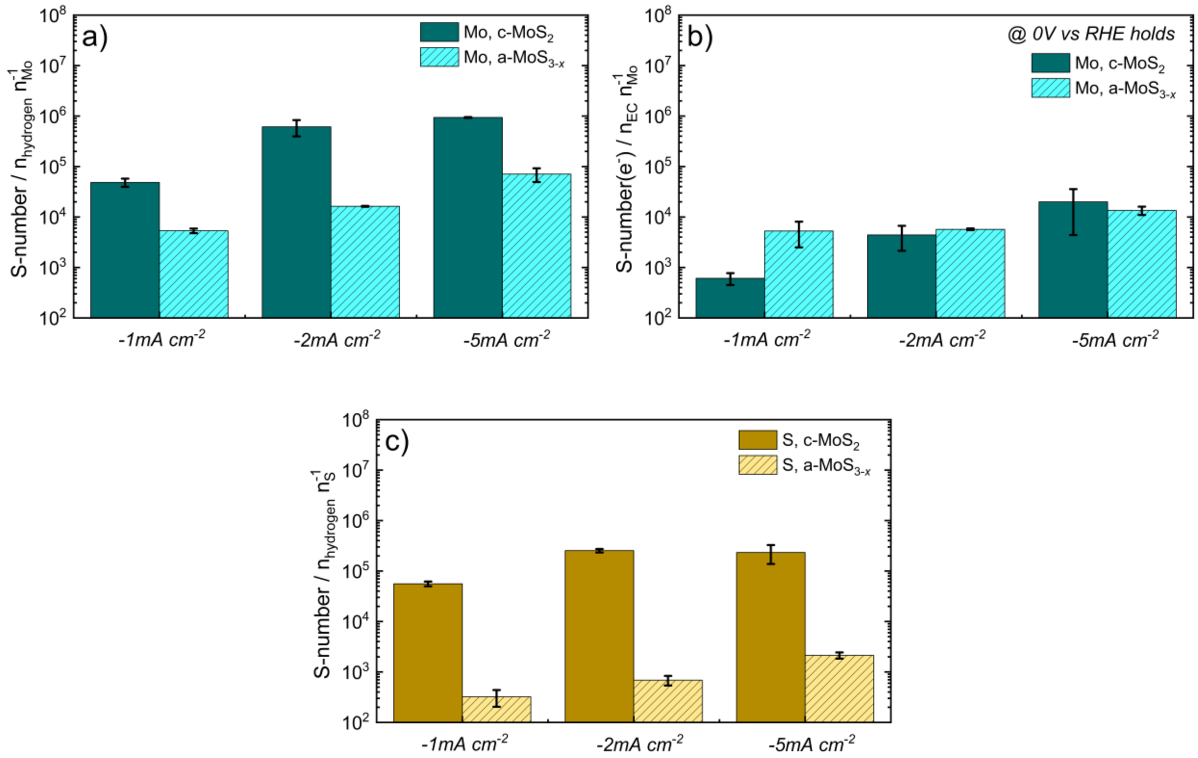

Compilation of S-numbers obtained for c-MoS_2 and a-MoS_{3-x} during sequential galvanostatic holds for a-b) Mo and c) S under HER conditions (left) and S-numbers(e^-) during 0V vs. RHE holds (right).

Figure S19. Compiled loading-normalized dissolution obtained during sequential galvanostatic holds

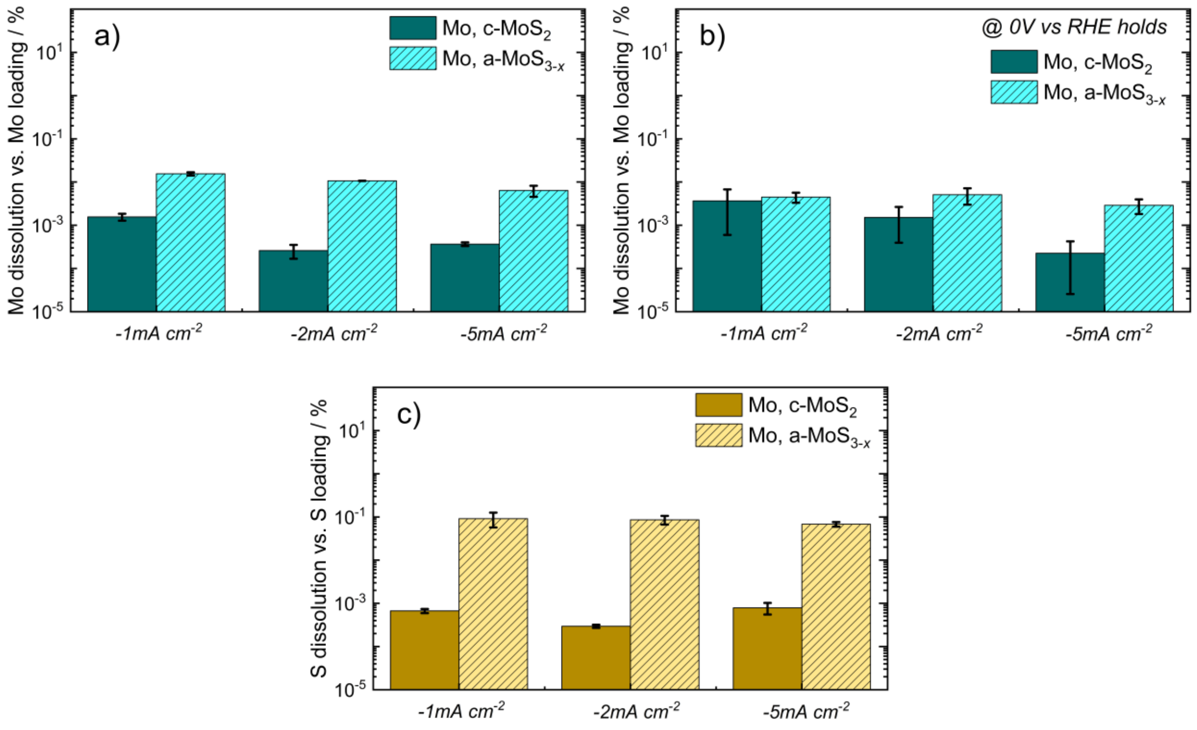

Compilation of loading-normalized integrated dissolution of a-b) Mo and c) S for c-MoS_2 and a-MoS_{3-x} during sequential galvanostatic holds under HER conditions (a,c) and during 0V vs. RHE holds (b).

Figure S20. Online ICP-MS data during sequential galvanostatic holds for [Mo_3S_{13}]-based catalysts

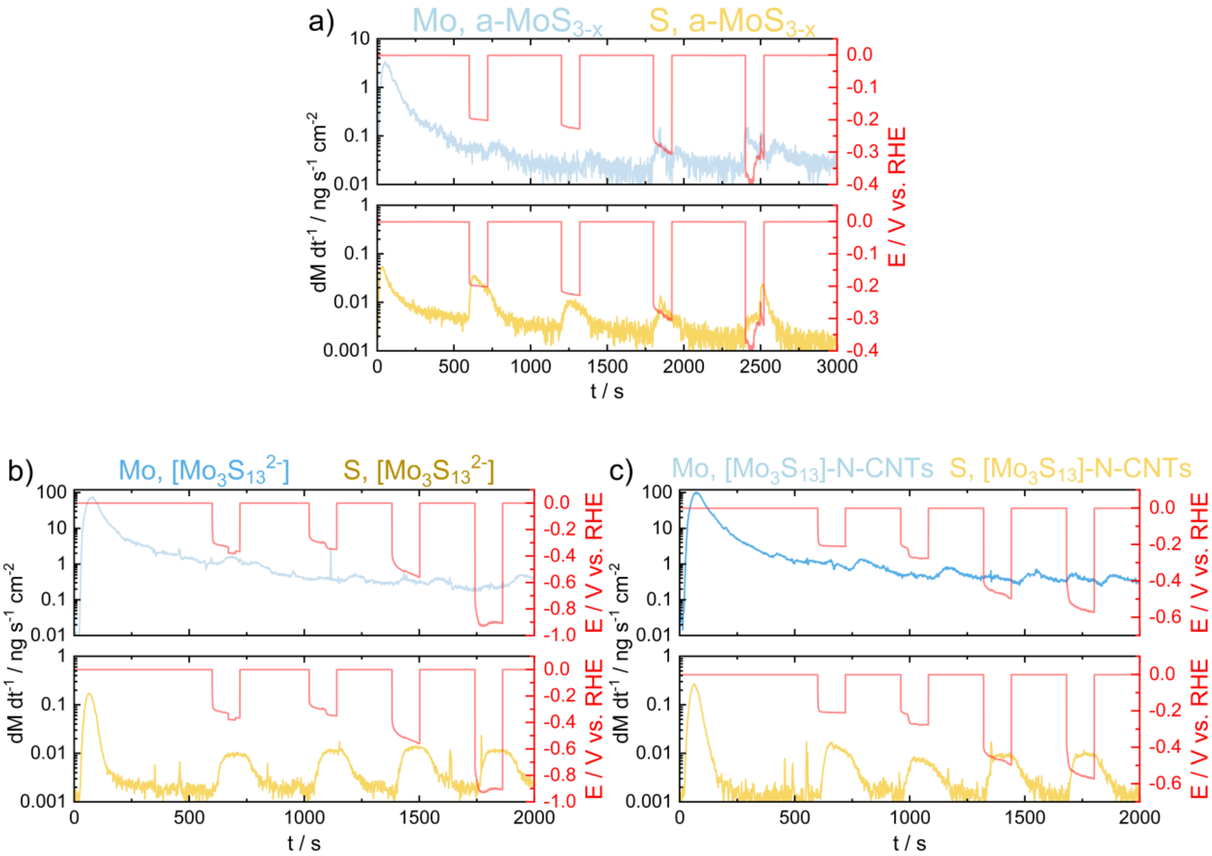

Online ICP-MS dissolution data obtained for a) a-MoS_{3-x}, b) $[Mo_3S_{13}]^{2-}$ and c) [Mo_3S_{13}]-N-CNTs during sequential galvanostatic holds with increasing cathodic geometric current density (j_{geom}= -1, -2, -5 , -10 mA cm^{-2}, 2 min) values for Mo (blue) and S (yellow). Potentiostatic holds at 0 V_{RHE} (8 mins) are employed here to resolve dissolution signals at 0 V vs. RHE.

Figure S21. Compiled S-numbers obtained during sequential galvanostatic holds for $[Mo_3S_{13}]$-based catalysts

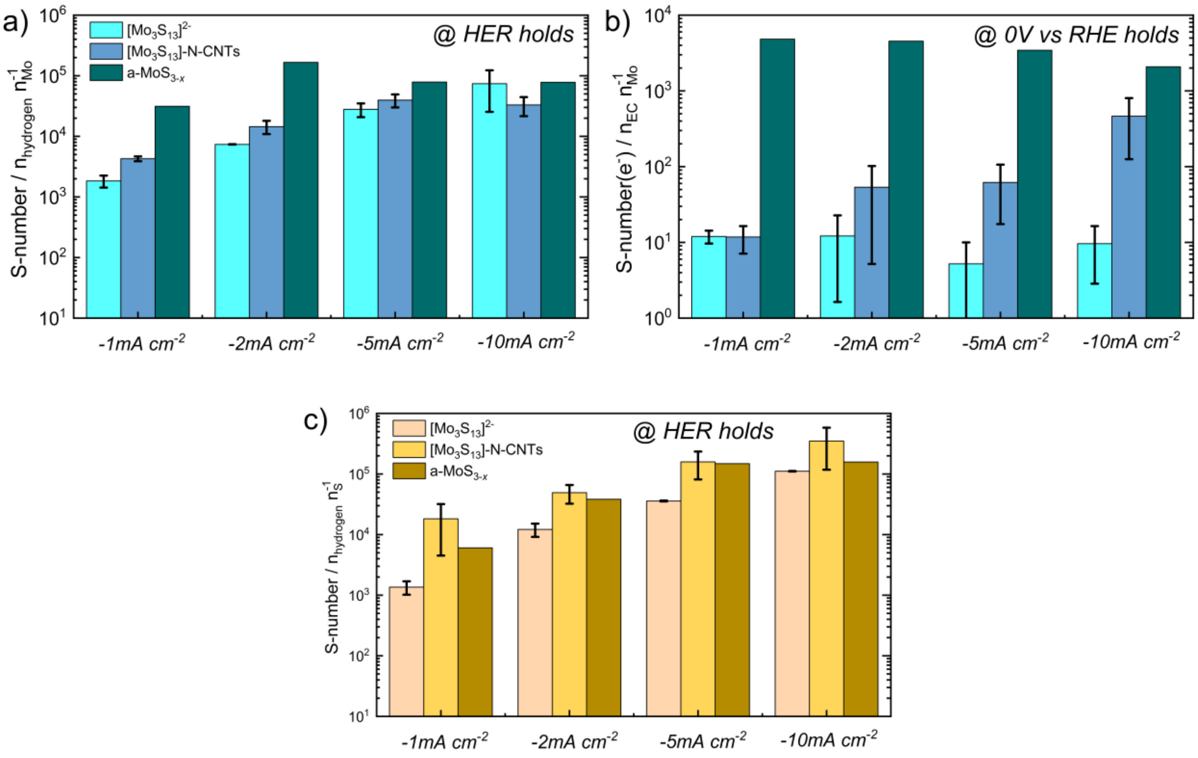

Compilation of S-numbers obtained for a-$MoS_{3\text{-}x}$, $[Mo_3S_{13}]^{2-}$ and $[Mo_3S_{13}]$-N-CNTs during sequential galvanostatic holds for a) Mo and b) S under HER conditions (a,c) and S-numbers(e^-) during 0V vs. RHE holds (b).

Figure S22. Loading-normalized integrated dissolution obtained during sequential galvanostatic holds for [Mo_3S_{13}]-based catalysts

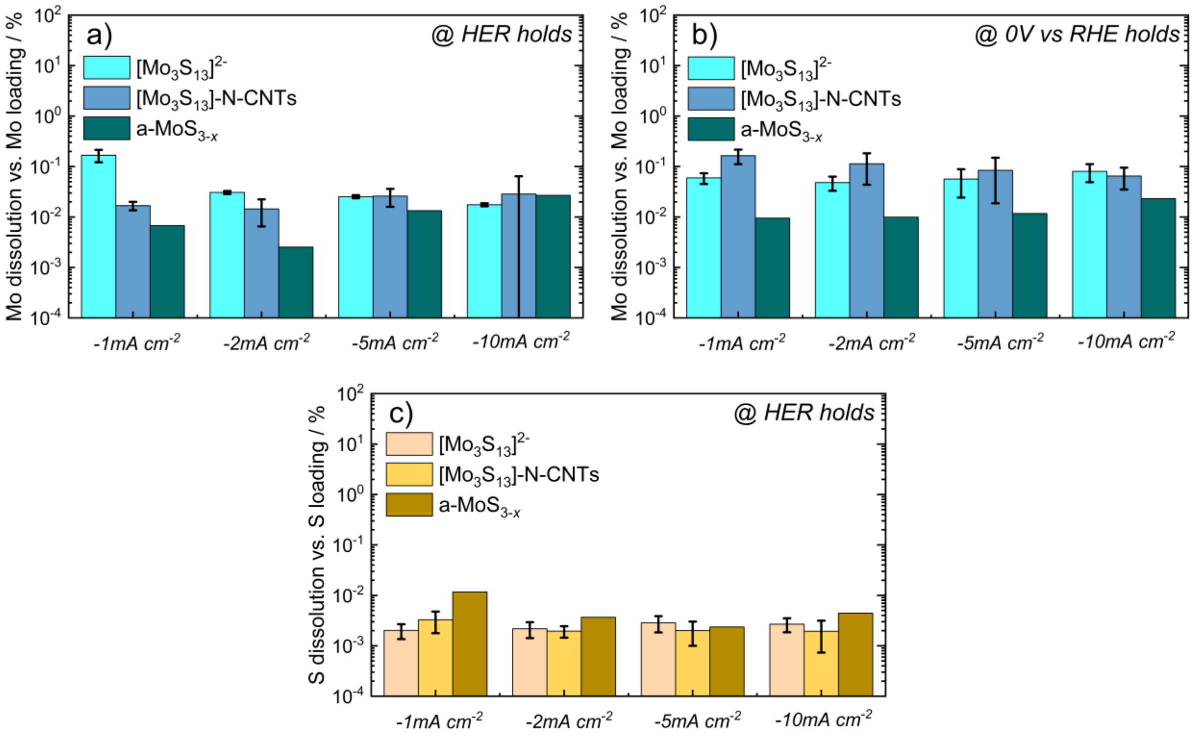

Compilation of loading-normalized integrated dissolution of a-b) Mo and c) S obtained for a-MoS_{3-x}, [Mo_3S_{13}]$^{2-}$ and [Mo_3S_{13}]-N-CNTs during sequential galvanostatic holds under a,c) HER conditions b) and during 0V vs. RHE holds.

Figure S23. Electrochemical data obtained for long-term, high-current HER measurements

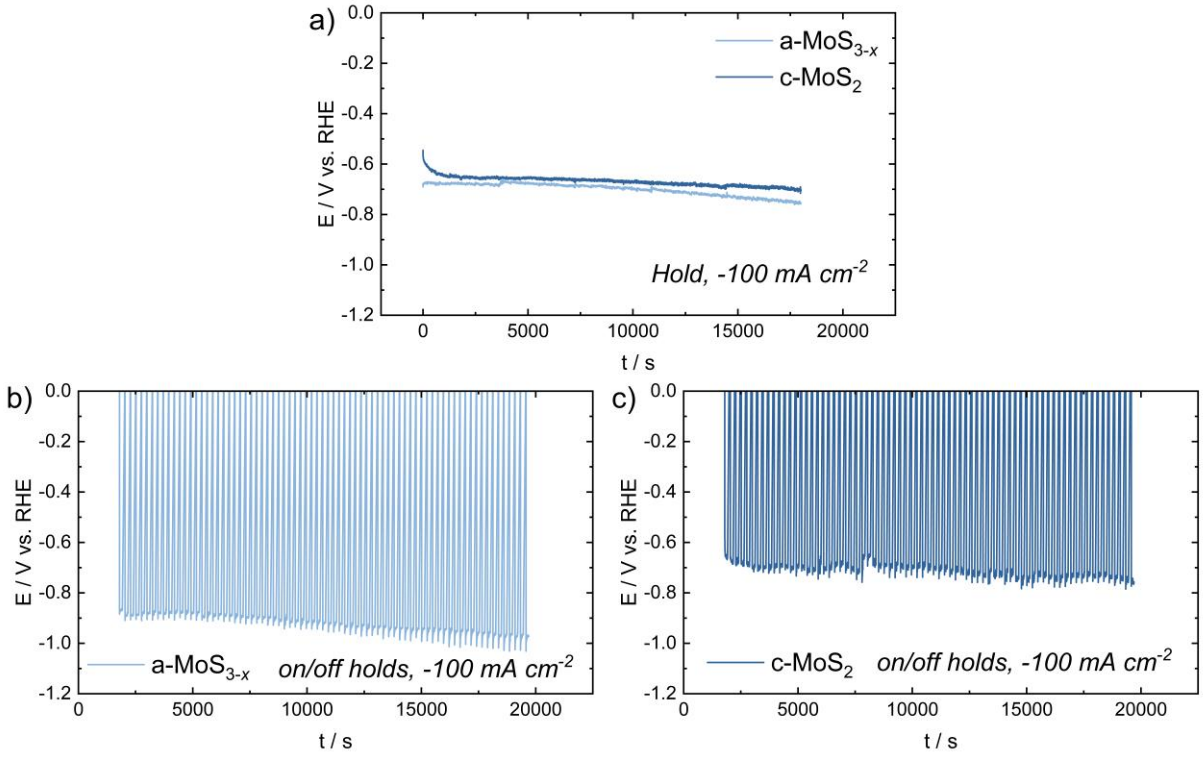

Compilation of potential vs. time curves obtained for a-$MoS_{3\text{-}x}$ (light blue) and c-MoS_2 (dark blue) during H-cell measurements consisting of a) 5 hours constant galvanostatic hold at -100 mA cm^{-2} and b-c) 5 hours undergoing start-up/shutdown HER holds alternating from -100 mA cm^{-2} to 0 V vs. RHE (bottom panel).

Figure S24. Total and loading-normalized dissolution obtained for long-term, high-current HER measurements

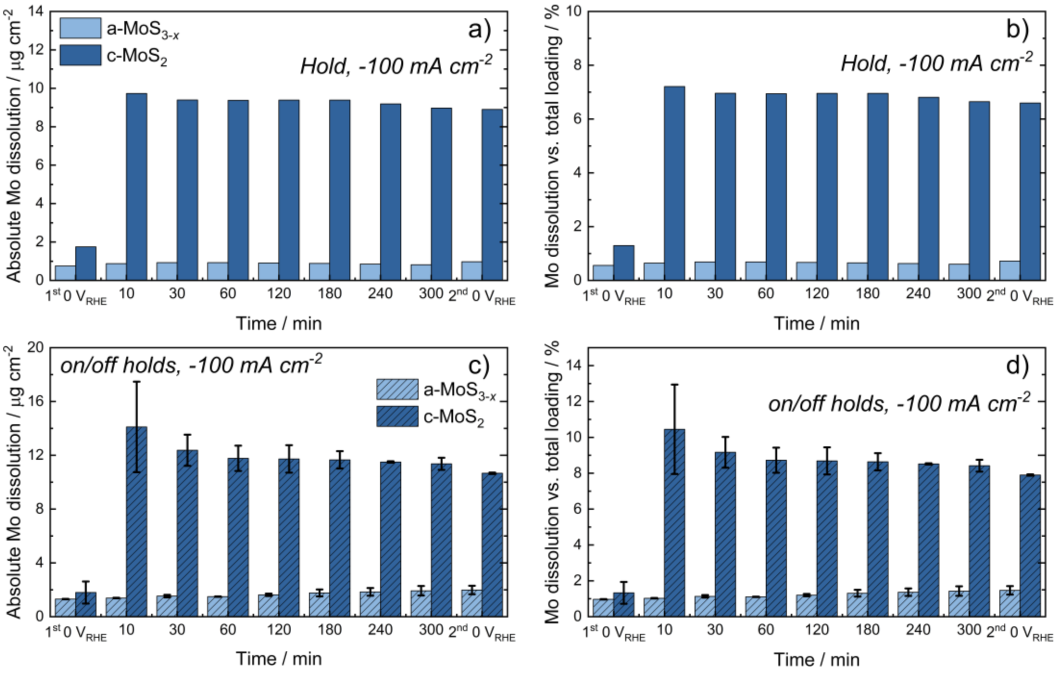

Compilation of a,c) total and b-d) loading-normalized integrated dissolution of Mo for a-MoS_{3-x} (light blue) and c-MoS_2 (dark blue) obtained after H-cell measurements consisting of 5 hours constant galvanostatic hold at -100 mA cm^{-2} (solid columns) and after 5 hours undergoing start-up/shutdown HER holds alternating from -100 mA cm^{-2} to 0 V vs. RHE (dashed columns).

Figure S25. Physicochemical characterization of MoS_x thin films prepared for long-term, high-current HER measurements

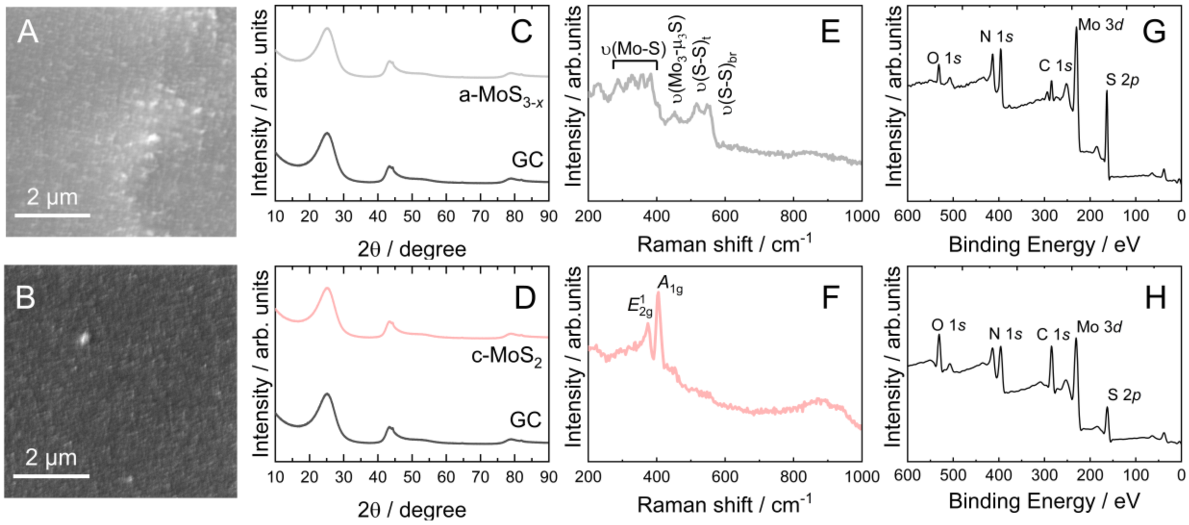

Physicochemical characterization of the pristine anodically (A, C, E, G) and cathodically (B, D, F, H) electrodeposited MoS_x thin films employed for long-term stability testing. A, B) Show the scanning electron micrographs, C, D) the XRD diffractograms including the glassy carbon substrate (GC) as reference for the backing electrode contribution, E, F) the Raman spectra, and G, H) the XPS survey spectra.

Figure S26. Physicochemical characterization of c-MoS2 thin films before/after long-term, high-current HER measurements

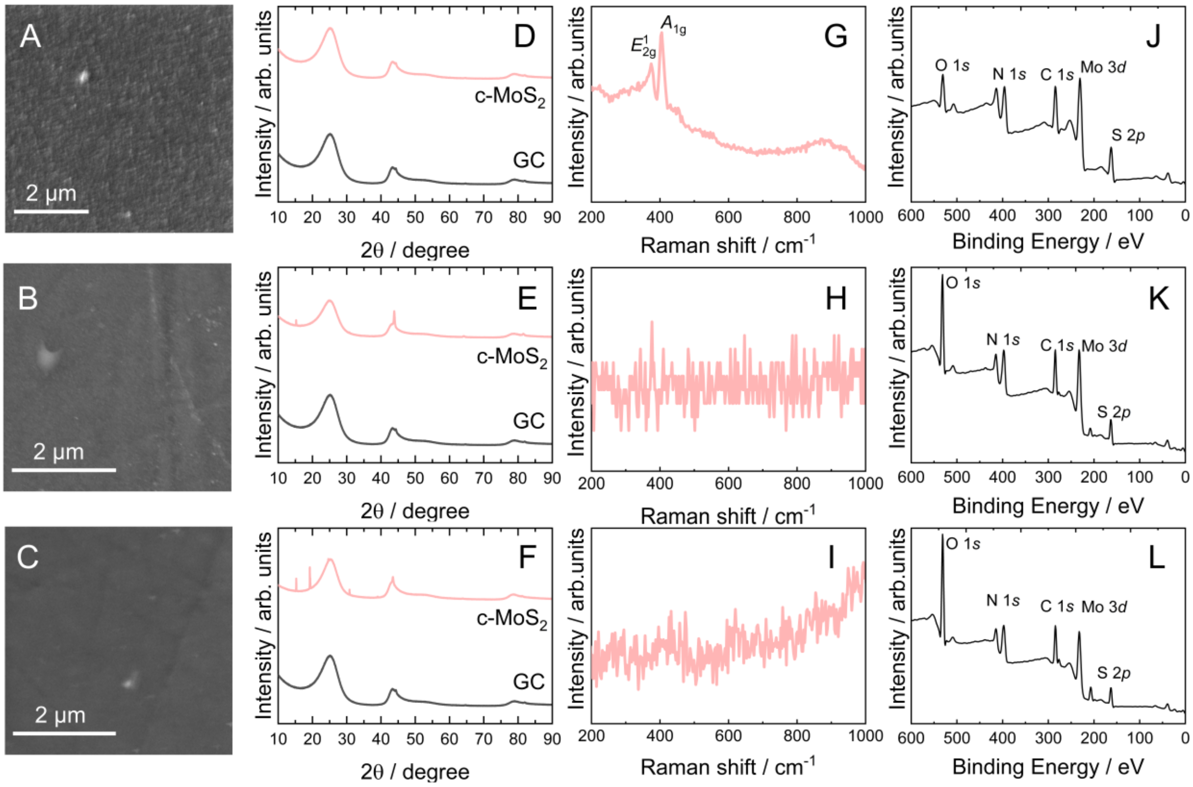

Physicochemical characterization of cathodically electrodeposited MoS_x thin films employed for long-term stability testing: pristine (A, D, G, J), after 5 hours constant galvanostatic hold at -100 mA cm^{-2} (B, E, H, K) and after 5 hours undergoing start-up/shutdown HER holds alternating from -100 mA cm^{-2} to 0 V vs. RHE (C, F, I, L). A-C) Show the scanning electron micrographs, D-F) the XRD diffractograms including the glassy carbon substrate (GC) as reference for the backing electrode contribution, G-I) the Raman spectra, and J-L) the XPS survey spectra.

Figure S27. Ex-situ XPS data on c-MoS_2 thin films before/after long-term, high-current HER measurements

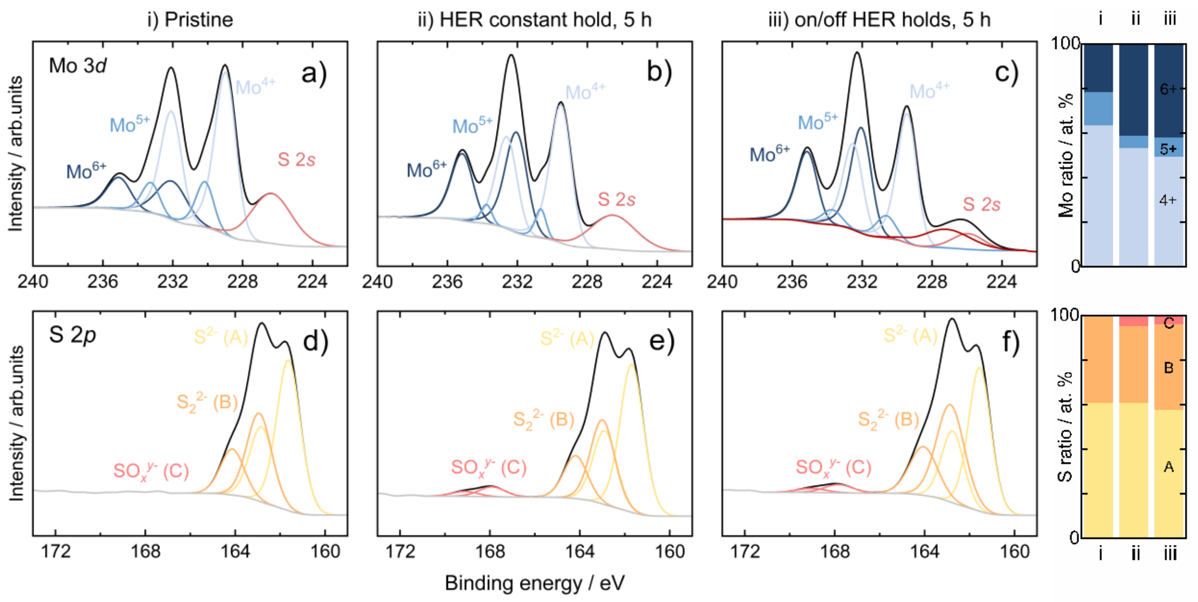

High-resolution Mo 3*d* (a-c) and S 2*p* (d-f) XPS spectra of pristine c-MoS_2 thin films before electrochemistry (I; a,d), after 5 hours constant galvanostatic hold at -100 mA cm^{-2} (II; b,e), and after 5 hours undergoing start-up/shutdown HER holds alternating from -100 mA cm^{-2} to 0 V vs. RHE (III; c,f). Labels: cumulative peak fit (black), Mo^{4+} $3d_{5/2:3/2}$ (light blue), $Mo^{a}O_{b}S_{c}$ $3d_{5/2:3/2}$ (blue), Mo^{6+} $3d_{5/2:3/2}$ (dark blue), S $2p_{3/2:1/2}$ (S^{2-}, yellow), S $2p_{3/2:1/2}$ (S_2^{2-}, orange) and S $2p_{3/2:1/2}$ (SO_x^{y-}, red).

Figure S28. Physicochemical characterization of a-MoS_{3-x} thin films before/after long-term, high-current HER measurements

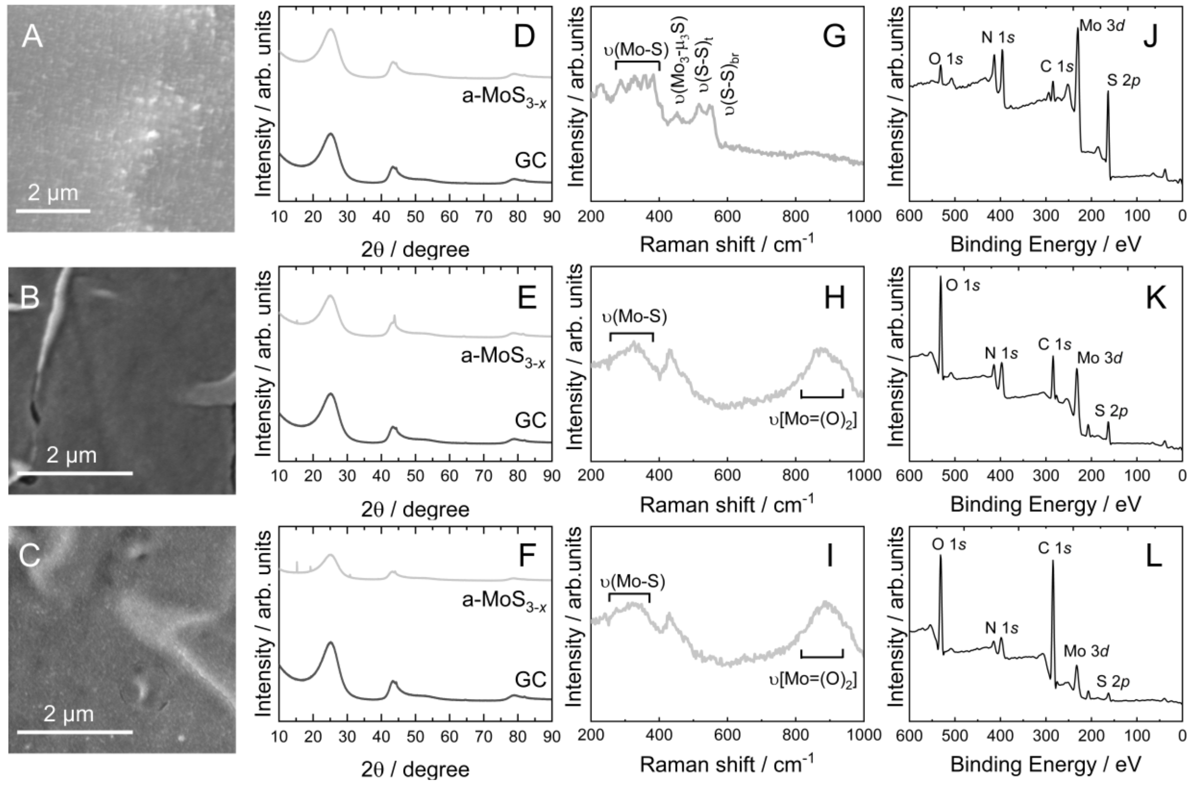

Physicochemical characterization of anodically electrodeposited MoS_x thin films employed for long-term stability testing: pristine (A, D, G, J), after 5 hours constant galvanostatic hold at -100 mA cm^{-2} (B, E, H, K) and after 5 hours undergoing start-up/shutdown HER holds alternating from -100 mA cm^{-2} to 0 V vs. RHE (C, F, I, L). A-C) Show the scanning electron micrographs, D-F) the XRD diffractograms including the glassy carbon substrate (GC) as reference for the backing electrode contribution, G-I) the Raman spectra, and J-L) the XPS survey spectra.

Figure S29. Ex-situ XPS data on a-MoS_{3-x} thin films before/after long-term, high-current HER measurements

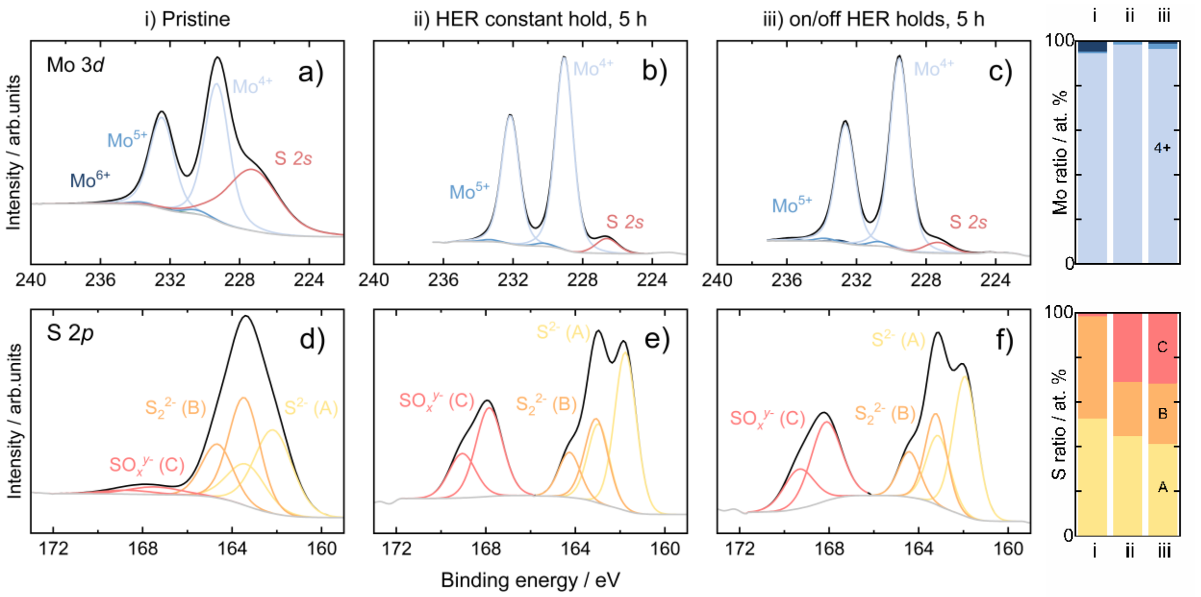

High-resolution Mo 3*d* (a-c) and S 2*p* (d-f) XPS spectra of pristine a-MoS_{3-x} thin films before electrochemistry (I; a,d), after 5 hours constant galvanostatic hold at -100 mA cm^{-2} (II; b,e), and after 5 hours undergoing start-up/shutdown HER holds alternating from -100 mA cm^{-2} to 0 V vs. RHE (III; c,f). Labels: cumulative peak fit (black), Mo^{4+} $3d_{5/2:3/2}$ (light blue), $Mo^{a}O_{b}S_{c}$ $3d_{5/2:3/2}$ (blue), Mo^{6+} $3d_{5/2:3/2}$ (dark blue), S $2p_{3/2:1/2}$ (S^{2-}, yellow), S $2p_{3/2:1/2}$ (S_2^{2-}, orange) and S $2p_{3/2:1/2}$ (SO_x^{y-}, red).

HER pre-conditioning	MoS_x catalyst	Peak current density, first cycle / mA cm^{-2}	Peak current density, last cycle / mA cm^{-2}
100 CVs,<br>0 to -0.25 V vs. RHE	c-MoS_2<br>a-MoS_{3-x}	-0.60±0.08<br>-0.8±0.4	-0.48±0.03<br>-7.6±0.6
150 CVs,<br>0 to -0.2 V vs. RHE	$[Mo_3S_{13}]^{2-}$<br>Mo_3S_{13}-N-CNT<br>a-MoS_{3-x}	-0.137±0.005<br>-1.22±0.02<br>-0.7±0.2	-0.09±0.006<br>-2.09±0.05<br>-1±0.3

Table S1. Compilation of peak current densities recording during potentiodynamic cycling at the first and last cycles.

HER pre-conditioning	MoS_x catalyst	S-to-Mo ratio, pre-HER theoretical (XPS)	S-to-Mo ratio, ICP-MS post-HER	S-to-Mo ratio, post-HER XPS
100 CVs, 0 to -0.25 V vs. RHE	c-MoS_2	2 (1.5)	2.0035±0.0007	-
	a-$MoS_{3\text{-}x}$	3 (2.8)	2.995±0.007	-
150 CVs, 0 to -0.2 V vs. RHE	$[Mo_3S_{13}]^{2-}$	4.333 (4.151)	4.3435±0.0001	2.75
	Mo_3S_{13}-N-CNT	4.333 (4.193)	4.525±0.004	1.58
	a-$MoS_{3\text{-}x}$	3 (2.8)	-	-

Table S2. Compilation of S-to-Mo ratios expected for all MoS_x catalysts in their pristine state, and values obtained after a back-of-the-envelope calculation based on Mo/S relative dissolution versus initial loadings detected downstream after completion of the start-up/shut-down HER stress tests, and post-mortem ex-situ XPS.

HER testing	MoS_x catalyst	Loading-normalized total Mo loss (%)	Final Mo S-numbers, H-cell
Pristine	c-MoS_2	-	-
-100 mA cm^{-2}, constant 5 h hold		6.59	9.96×10^4
-100 mA cm^{-2}, on-off 5 h hold		7.90	3.89×10^4
Ratio, constant vs. on-off		0.83	2.60
Pristine	a-$MoS_{3\text{-}x}$	-	-
-100 mA cm^{-2}, constant 5 h hold		0.72	1.10×10^6
-100 mA cm^{-2}, on-off 5 h hold		1.47	2.33×10^5
Ratio, constant vs. on-off		0.49	4.72
		5 h hold	on-off 5 h hold
c-MoS_2-to- a-$MoS_{3\text{-}x}$ ratio, loading-normalized Mo loss		9.2	5.4
a-$MoS_{3\text{-}x}$-to-c-MoS_2 ratio, final Mo S-numbers, H-cell		11.0	6.0

Table S3. Compilation of loading-normalized total dissolution and final S-numbers from Mo quantification in a-$MoS_{3\text{-}x}$ and c-MoS_2 thin films during long-term HER testing. Values obtained after 5 hours constant galvanostatic hold at -100 mA cm^{-2} and after 5 hours undergoing start-up/shutdown HER holds alternating from -100 mA cm^{-2} to 0 V vs. RHE, using an H-cell configuration. Data is graphically presented in Figures 9 and S24.

HER testing	MoS_x catalyst	S-to-Mo ratio, pre-HER theoretical (XPS)	S-to-Mo ratio, post-HER XPS
Pristine<br>-100 mA cm^{-2}, 5 h hold<br>-100 mA cm^{-2}, on-off 5 h hold	c-MoS_2	2 (2.03)	 1.47<br>1.35
Pristine<br>-100 mA cm^{-2}, 5 h hold<br>-100 mA cm^{-2}, on-off 5 h hold	a-MoS_{3-x}	4.333 (4.36)	 0.38<br>0.36

Table S4. Compilation of S-to-Mo ratios expected for a-MoS_{3-x} and c-MoS_2 thin films in their pristine state, and values obtained after after 5 hours constant galvanostatic hold at -100 mA cm^{-2} and after 5 hours undergoing start-up/shutdown HER holds alternating from -100 mA cm^{-2} to 0 V vs. RHE, using post-mortem ex-situ XPS on samples presented in Figures S27 and S29.

References

1. Merki D, Fierro S, Vrubel H, Hu X. Amorphous molybdenum sulfide films as catalysts for electrochemical hydrogen production in water. *Chem Sci* **2**, 1262-1267 (2011).

2. Bélanger D, Laperriére G, Marsan B. The electrodeposition of amorphous molybdenum sulfide. *Journal of Electroanalytical Chemistry* **347**, 165-183 (1993).

3. Redman DW, Rose MJ, Stevenson KJ. Electrodeposition of Amorphous Molybdenum Chalcogenides from Ionic Liquids and Their Activity for the Hydrogen Evolution Reaction. *Langmuir* **33**, 9354-9360 (2017).

4. Chia X, Sutrisnoh NAA, Pumera M. Tunable Pt-MoS (x) Hybrid Catalysts for Hydrogen Evolution. *ACS Appl Mater Interfaces* **10**, 8702-8711 (2018).

5. Wang HW, Skeldon P, Thompson GE. XPS studies of MoS2 formation from ammonium tetrathiomolybdate solutions. *Surface and Coatings Technology* **91**, 200-207 (1997).

6. Vrubel H, Hu X. Growth and Activation of an Amorphous Molybdenum Sulfide Hydrogen Evolving Catalyst. *ACS Catalysis* **3**, 2002-2011 (2013).

7. Weber T, Muijsers JC, Niemantsverdriet JW. Structure of Amorphous MoS3. *Journal of Physical Chemistry* **99**, 9194-9200 (1995).

8. Morales-Guio CG, Hu X. Amorphous molybdenum sulfides as hydrogen evolution catalysts. *Acc Chem Res* **47**, 2671-2681 (2014).

9. Ting LRL, Deng Y, Ma L, Zhang Y-J, Peterson AA, Yeo BS. Catalytic Activities of Sulfur Atoms in Amorphous Molybdenum Sulfide for the Electrochemical Hydrogen Evolution Reaction. *ACS Catalysis* **6**, 861-867 (2016).

10. Mabayoje O, *et al.* Electrodeposition of MoS(x) Hydrogen Evolution Catalysts from Sulfur-Rich Precursors. *ACS Appl Mater Interfaces* **11**, 32879-32886 (2019).

11. Iffelsberger C, Pumera M. High resolution electrochemical additive manufacturing of microstructured active materials: case study of MoSxas a catalyst for the hydrogen evolution reaction. *Journal of Materials Chemistry A* **9**, 22072-22081 (2021).

12. Speck FD, *et al.* Atomistic Insights into the Stability of Pt Single-Atom Electrocatalysts. *J Am Chem Soc* **142**, 15496-15504 (2020).

13. Brookins DG. *Eh-pH Diagrams for Geochemistry*. Springer-Verlag (1987).

14. Wang Z, *et al.* Origins of the Instability of Nonprecious Hydrogen Evolution Reaction Catalysts at Open-Circuit Potential. *ACS Energy Letters* **6**, 2268-2274 (2021).

15. Holzapfel PKR, *et al.* Fabrication of a Robust PEM Water Electrolyzer Based on Non-Noble Metal Cathode Catalyst: [Mo(3) S(13)](2-) Clusters Anchored to N-Doped Carbon Nanotubes. *Small* **16**, e2003161 (2020).

16. Nguyen DN, Nguyen LN, Nguyen PD, Thu TV, Nguyen AD, Tran PD. Crystallization of Amorphous Molybdenum Sulfide Induced by Electron or Laser Beam and Its Effect on H2-Evolving Activities. *The Journal of Physical Chemistry C* **120**, 28789-28794 (2016).

17. Escalera-López D, Lou Z, Rees NV. Benchmarking the Activity, Stability, and Inherent Electrochemistry of Amorphous Molybdenum Sulfide for Hydrogen Production. *Advanced Energy Materials* **9**, (2019).

18. Bonde J, Moses P, Jaramillo TF, Norskov J, Chorkendorff I. Hydrogen evolution on nano-particulate transition metal sulfides. *Faraday Discuss* **140**, 219-231 (2009).

19. Bau JA, Emwas A-H, Nikolaienko P, Aljarb AA, Tung V, Rueping M. Mo3+ hydride as the common origin of H2 evolution and selective NADH regeneration in molybdenum sulfide electrocatalysts. *Nature Catalysis* **5**, 397-404 (2022).

20. Tran PD, *et al*. Coordination polymer structure and revisited hydrogen evolution catalytic mechanism for amorphous molybdenum sulfide. *Nat Mater* **15**, 640-646 (2016).
